# Immunoinformatics-Based Designing of a Multi-Epitope Chimeric Vaccine From Multi-Domain Outer Surface Antigens of *Leptospira*

**DOI:** 10.3389/fimmu.2021.735373

**Published:** 2021-11-30

**Authors:** Pankaj Kumar, Surabhi Lata, Umate Nachiket Shankar, Mohd. Akif

**Affiliations:** Laboratory of Structural Biology, Department of Biochemistry, School of Life Sciences, University of Hyderabad, Hyderabad, India

**Keywords:** *Leptospira interrogans*, antigenic epitope, outer surface antigen, vaccine, *Leptospira* immunoglobulin-like protein, subunit vaccine, immunoinformatics

## Abstract

Accurate information on antigenic epitopes within a multi-domain antigen would provide insights into vaccine design and immunotherapy. The multi-domain outer surface *Leptospira* immunoglobulin-like (Lig) proteins LigA and LigB, consisting of 12–13 homologous bacterial Ig (Big)-like domains, are potential antigens of *Leptospira interrogans*. Currently, no effective vaccine is available against pathogenic *Leptospira*. Both the humoral immunity and cell-mediated immunity of the host play critical roles in defending against *Leptospira* infection. Here, we used immunoinformatics approaches to evaluate antigenic B-cell lymphocyte (BCL) and cytotoxic T-lymphocyte (CTL) epitopes from Lig proteins. Based on certain crucial parameters, potential epitopes that can stimulate both types of adaptive immune responses were selected to design a chimeric vaccine construct. Additionally, an adjuvant, the mycobacterial heparin-binding hemagglutinin adhesin (HBHA), was incorporated into the final multi-epitope vaccine construct with a suitable linker. The final construct was further scored for its antigenicity, allergenicity, and physicochemical parameters. A three-dimensional (3D) modeled construct of the vaccine was implied to interact with Toll-like receptor 4 (TLR4) using molecular docking. The stability of the vaccine construct with TLR4 was predicted with molecular dynamics simulation. Our results demonstrate the application of immunoinformatics and structure biology strategies to develop an epitope-specific chimeric vaccine from multi-domain proteins. The current findings will be useful for future experimental validation to ratify the immunogenicity of the chimera.

## Introduction

Leptospirosis is categorized as an emerging and neglected tropical zoonotic disease worldwide. It is considered a public health problem globally, with an estimated 1 million leptospirosis cases reported each year, causing deaths of around 60,000 (1–3). The infection usually shows symptoms such as headache, chills, illness, and muscle aches, and a more severe form of disease is associated with multi-organ failure known as Weil’s disease. The causative agent, pathogenic *Leptospira* spp., includes more than 270 antigenically diverse serovars (4). There is a lack of proper therapeutics and effective vaccines against pathogenic *Leptospira*. Hence, an advance in the development of new therapeutics and effective subunit vaccines is warranted.

The vaccines available at present are either based on inactivated whole cell or membrane preparations from pathogenic *Leptospira* species. These are associated with severe side effects and do not provide cross protection among the pathogenic *Leptospira* species (5, 6). In order to control the disease, several virulent factors involved in the pathogenesis of *Leptospira* are being investigated as both therapeutic targets and vaccine candidates. Outer membrane/surface proteins (OMPs) from pathogenic *Leptospira* play a major part in establishing infection and are being explored as attractive vaccine targets as most of them are conserved across the serovars and can be recognized by the host immune response in the early phase of infection (7). Currently, *Leptospira* immunoglobulin-like (Lig) proteins are reported to be the most antigenic and as attractive vaccine candidates (8).

The family of Lig proteins, present exclusively in pathogenic species and composed of LigA and LigB, with 13 and 12 homologous extracellular immunoglobulin (Ig)-like repeated domains, respectively, plays an important role in virulence (8–10). These homologous domains possess similarity in structure to the adhesin domains from enterobacterial pathogens (11). The family of Lig proteins binds to host extracellular matrix (ECM) components and helps pathogens to invade, as well as helping in host tissue colonization (9, 12) In addition, Lig proteins are known to bind to the complement factors in order to evade the innate immunity and establish infection (13). It has been reported that Lig proteins could induce significant protection against lethality in a hamster challenge model (11, 14, 15). Moreover, in a mouse model, ~90% protection was induced by Lig proteins (16). The ability of Lig proteins to bind varieties of host factors and attribute toward immune induction made it possible to consider them as putative virulent factors and the most important vaccine candidates identified to date (17). Recently, it has been shown that antigenic motifs in a single-domain chimeric Ig-like fold generate enhanced leptospiral protection compared to whole Lig proteins (18).

The Lig family of proteins is composed of Bacterial Immunoglobulin-like (Big), multi-domain proteins. It is thought that its immunogenic region may not be well accessible. Hence, knowledge of the immunogenic region/epitope will provide opportunities for further improvements in the vaccine efficacy of the Lig family proteins through rational design. Several methods that can be used to instigate the immunogenic region or epitope of the Lig family of proteins are available. Most of these methods are expensive and time-consuming for vaccine development. However, with the availability of several computational algorithms, the identification of immunogenic regions or epitopes has become an easy task and has expedited the vaccine development research (19). There are various successful efforts toward epitope-based vaccines against diverse pathogens such as HIV (20), influenza virus (21), and hepatitis B and C viruses. Similar approaches have been used to identify potential epitopes against various antigens of coronavirus disease 2019 (COVID-19) (22, 23). The two important arms that counter and eliminate the pathogens are the humoral and the cell-mediated immune response. Hence, the identification of B-cell lymphocyte (BCL) and cytotoxic T lymphocyte (CTL) epitopes present on proteins is an important step for the design of epitope-based engineered vaccines (24).

In the present study, an immunoinformatics approach was applied for the comprehensive evaluation of antigenic epitopes present on the Lig family of proteins from *Leptospira interrogans*. Various T-cell epitopes were generated and their binding interactions with the major histocompatibility complex (MHCs) were analyzed. Subsequently, structural and continuous B-cell epitopes were also predicted. In order to increase the confidentiality of prediction, three different tools were used to select common and overlapping epitopes. These epitopes were fused together with suitable linkers and incorporated with an adjuvant to generate a multi-epitope chimeric vaccine construct.

## Methodology

### Protein Sequence Retrieval

Full amino acid sequences of LigA (accession no. ACH89909.1) and LigB (accession no. AAP74956.1) were downloaded from NCBI (https://www.ncbi.nlm.nih.gov/protein) in FASTA format. The sequences of individual Ig-like domains were separated from the full-length Lig sequences and saved separately. All the prepared sequences of Ig-like domains were subsequently subjected to antigenic epitope prediction.

### Prediction of BCL Epitopes

Three different tools were used to predict the conformational and linear BCL epitopes. Since structural information for conformational epitope prediction was required, all individual Ig-like domains (seven variable domains from LigA and all 12 domains from LigB) of the Lig proteins were modeled using the online server I-TASSER (Iterative Threading Assembly Refinement) (https://zhanglab.dcmb.med.umich.edu/I-TASSER/) (25). I-TASSER took the terminal Ig-like domain (LigB12) NMR structure as a template to model other individual Ig-like domains (10). Using the online tool GalaxyRefine (http://galaxy.seoklab.org/cgi-bin/submit.cgi?type=REFINE) (26), the models were further refined and energy minimized using the Swiss-PdbViewer (SPDBV) tool (https://spdbv.vital-it.ch/) (27). The quality of the models was checked by ProSA-web (https://prosa.services.came.sbg.ac.at/prosa.php) (28) and validated using Ramachandran plot analysis (https://servicesn.mbi.ucla.edu/PROCHECK/) (29). Three different online tools—DiscoTope2.0 (http://www.cbs.dtu.dk/services/DiscoTope/) (30), ElliPro (http://tools.iedb.org/ellipro/) (31), and BEPro (http://pepito.proteomics.ics.uci.edu/) (32)—were employed for conformational epitope prediction, with the default parameters. In the case of the sequence-based linear BCL epitope prediction, ABCpred (https://webs.iiitd.edu.in/raghava/abcpred/) (33), BCPred (http://ailab-projects1.ist.psu.edu:8080/bcpred/) (34), and BepiPred 2.0 (http://www.cbs.dtu.dk/services/BepiPred/) (35) were used with the default parameters.

### CTL and HTL Epitope Prediction

Potential CTL antigenic epitopes were predicted from each Ig-like domain using three different servers with the default parameters: IEDB (http://tools.iedb.org/mhci/) (36), ProPred-I (https://webs.iiitd.edu.in/raghava/propred1/) (37), and NetMHC 4.0 (http://www.cbs.dtu.dk/services/NetMHC/) (36). The IEDB tool works on an artificial neural network (ANN) to cover 80 different MHC-I alleles, which included 36 human leukocyte antigen A (HLA-A) alleles, 34 HLA-B alleles, and 10 HLA-C alleles. Peptides that showed an IC_50_ value ≤250 nM were selected. ProPred-I uses a matrix-based method to predict potential epitopes by covering 47 MHC class I alleles. It also predicts proteasomal cleavage sites present on antigens. At a default threshold of 4%, the prediction showed equal sensitivity and specificity toward all the alleles; thus, this default threshold was selected for our analysis. NetMHC 4.0 uses an ANN algorithm to predict the 9-mer peptide by covering 81 HLA alleles belonging to the classes HLA-A, HLA-B, HLA-C, and HLA-E. Only strong binding peptides that showed percent rank lower than 0.5 were selected. In all cases, peptides binding to MHC-I have strong preference for nonamer peptides; thus, nonamer peptides with good antigenic scores were selected from each server.

For the prediction of HTL epitopes, three different tools, namely, ProPred (https://webs.iiitd.edu.in/raghava/propred/page2.html) (38), IEDB (http://tools.iedb.org/mhcii/) (39), and NetMHC-II 2.3 (http://www.cbs.dtu.dk/services/NetMHCII/) (40), were used. ProPred uses quantitative matrices to predict 9- to 18-amino acid length peptides by covering 51 HLA-DR alleles in MHC-II. The peptides with a threshold ≤3% were selected. The IEDB server uses a consensus approach that predicts 15-mer peptides using different available methods. Peptides showing an adjusted rank ≤1 were selected. NetMHC-II 2.3 uses an ANN algorithm to predict 15-mer peptides. Peptides showing percent rank ≤2 were selected. NetMHC-II 2.3 covers a total of 61 different alleles consisting of HLA-DR, HLA-DQ, HLA-DP, and seven mouse H2 class II alleles. Peptides that showed binding to three or more different alleles of MHC-I and MHC-II alleles were considered as promiscuous.

### Removal of Self-Peptides and Selection of IFN-γ Inducing Epitopes

The presence of self-peptides can induce autoimmune responses. To overcome this problem, the peptides that showed overlapping with human peptides were removed using the ProteoMapper tool (http://www.peptideatlas.org/map/) (41) of the PeptideAtlas server. Interferon gamma (IFN-γ)-positive epitopes present among the HTL epitopes were selected using the IFNepitope server (https://webs.iiitd.edu.in/raghava/ifnepitope/predict.php) (42). IFN-γ epitopes play an important role in innate and in adaptive immune response by inducing T helper cells. The support vector machine (SVM) hybrid method was used to categorize the epitopes into IFN-γ and non-IFN-γ epitopes.

### Epitope Selection and Construction of the Multi-Epitope Vaccine

Common and overlapping BCL, CTL, and HTL epitopes predicted from all three servers were selected. Among them, the promiscuous, IFN-γ-positive, and non-self epitopes with the highest antigenic scores were shortlisted. Furthermore, the epitopes that were common and overlapping among all three lymphocytes were considered for the vaccine construct. The selected epitopes were linked with a suitable linker, such as EAAAK, GPGPG, and AAY, by giving sufficient space and flexibility among the peptides to fold correctly (43–45). Additionally, the mycobacterial heparin-binding hemagglutinin adhesin (HBHA) adjuvant is attached at the N-terminal to generate a multi-epitope chimeric vaccine construct (46).

### Immunoinformatics and Physicochemical Analysis of the Vaccine Construct

The constructed vaccine was analyzed for allergenicity using the AllerTop v. 2.0 tool (https://www.ddg-pharmfac.net/AllerTOP/) (47). This tool works on the auto cross-covariance (ACC) transformation of protein. Allergenicity of the vaccine was predicted using the amino acid sequence of the final multi-epitope vaccine. The antigenicity of the vaccine was analyzed using the VaxiJen 2.0 server (http://www.ddg-pharmfac.net/vaxijen/VaxiJen/) (48). The physicochemical parameters of the vaccine construct, such as the molecular weight, molar extinction coefficient, *in vivo* and *in vitro* half-life, and the grand average hydropathicity (GRAVY) and instability indices, were evaluated using the ProtParam tool (https://www.expasy.org/resources/protparam) (49).

### Prediction of the Secondary and Tertiary Structures

The secondary structure content of the multi-epitope vaccine construct was predicted with the PSIPRED server (http://bioinf.cs.ucl.ac.uk/psipred/) (50). PSIPRED uses a two-step ANN algorithm based on position-specific scoring matrices generated by PSI-BLAST. To model the 3D structure of the multi-epitope vaccine construct, the Robetta server (https://robetta.bakerlab.org/) (51) was used. The ProSA web server was used for further validation of the quality of the modeled structure.

### Molecular Docking of the Multi-Epitope Vaccine With Immune Receptor

For evaluating the interaction of the multi-epitope vaccine construct with the immune receptor, Toll-like receptor 4 (TLR4), the ClusPro server (https://cluspro.bu.edu/login.php) (52) was used with default parameters. The coordinate of TLR4 (PDBID: 3FXI) (53) was downloaded from RCSB (https://www.rcsb.org/), followed by removal of the non-TLR molecules. The monomeric form of TLR4 was used for docking with the vaccine construct. The complex between the two was generated by using three subsequent steps: rigid body docking, clustering of the lowest energy structure, and structural refinement. The best docked complex structure was selected on the basis of the maximum cluster size and the lowest energy score. The interaction in the complex was analyzed on LIGPLOT v2.2 and visualized on PyMOL (https://www.pymol.org/).

### Molecular Dynamics Simulation Studies

To study the stability of the interaction between the multi-epitope vaccine construct and TLR4, molecular dynamics (MD) simulation was carried out using the OPLS (Optimized Potentials for Liquid Simulations) all-atom force (54) field implemented in GROMACS 5.1.2 (55, 56). The SPC (simple point charge) was selected as a water model (57). The complex system was solvated in a cubic box with water molecules of 1.5 nm to the box wall from the surface of the protein. The system was further neutralized by adding Na^+^ counter ions. The steepest descent method for 25,000 steps was used to cut down the internal steric clashes until the largest force acting in the system was smaller than 1,000 kJ mol^−1^ nm^−1^. The complex was further equilibrated first in an NVT ensemble at 300 K for 50 ps using a modified V-rescale Berendsen thermostat with a time constant of 0.1 ps, followed by an NPT ensemble to 1 atm using the coupling method of Parrinello–Rahman with a time constant of 2 ps for 50 ps (58, 59). The complex was submitted for the final MD run for 100 ns at 300 K. The equations of motion were integrated with time steps of 2 fs and the coordinates were saved for every 2,500 time step (5 ps), which resulted in a total 4,000 frames for a 50-ns simulation. The particle mesh Ewald (PME) method with a real space cutoff of 10 Å was used for long-range electrostatic interactions (57). Constraints were applied for the bonds involving hydrogen by implementing the P-LINCS algorithm (60). A total of 20,000 frames were used for graph analysis from the production run. The root mean square deviations (RMSD), root mean square fluctuations (RMSF), and radius of gyration were respectively calculated using the gmx rms, gmx rmsf, and gmx gyrate commands implemented in the GROMACS. The graphs were plotted using XMGRACE.

### Immune Simulation

The immunogenic response of the multi-epitope chimeric vaccine construct was predicted using the online server C-ImmSim (https://150.146.2.1/C-IMMSIM/index.php?page=1) (61). Using the sequence information of the vaccine construct, both arms of immune responses were predicted. An immune simulation was performed with 100 simulation steps and one injection without lipopolysaccharide (LPS). The response recorded was analyzed further.

### *In Silico* Cloning

Finally, *in silico* cloning was done using a suitable plasmid containing a multi-epitope construct nucleotide sequence. The JCat server (http://www.jcat.de/) was utilized for codon optimization of the DNA sequence based on the *Escherichia coli* K12 strain (62). Percentage of GC ranging between 30 and 70 is treated as an optimal value. Cloning was done in pET28a(+) expression vector using SnapGene software at the *Bam*HI and *Hin*dIII restriction sites (63).

## Results

### Collection and Primary Sequence Analysis

**Figure 1:**
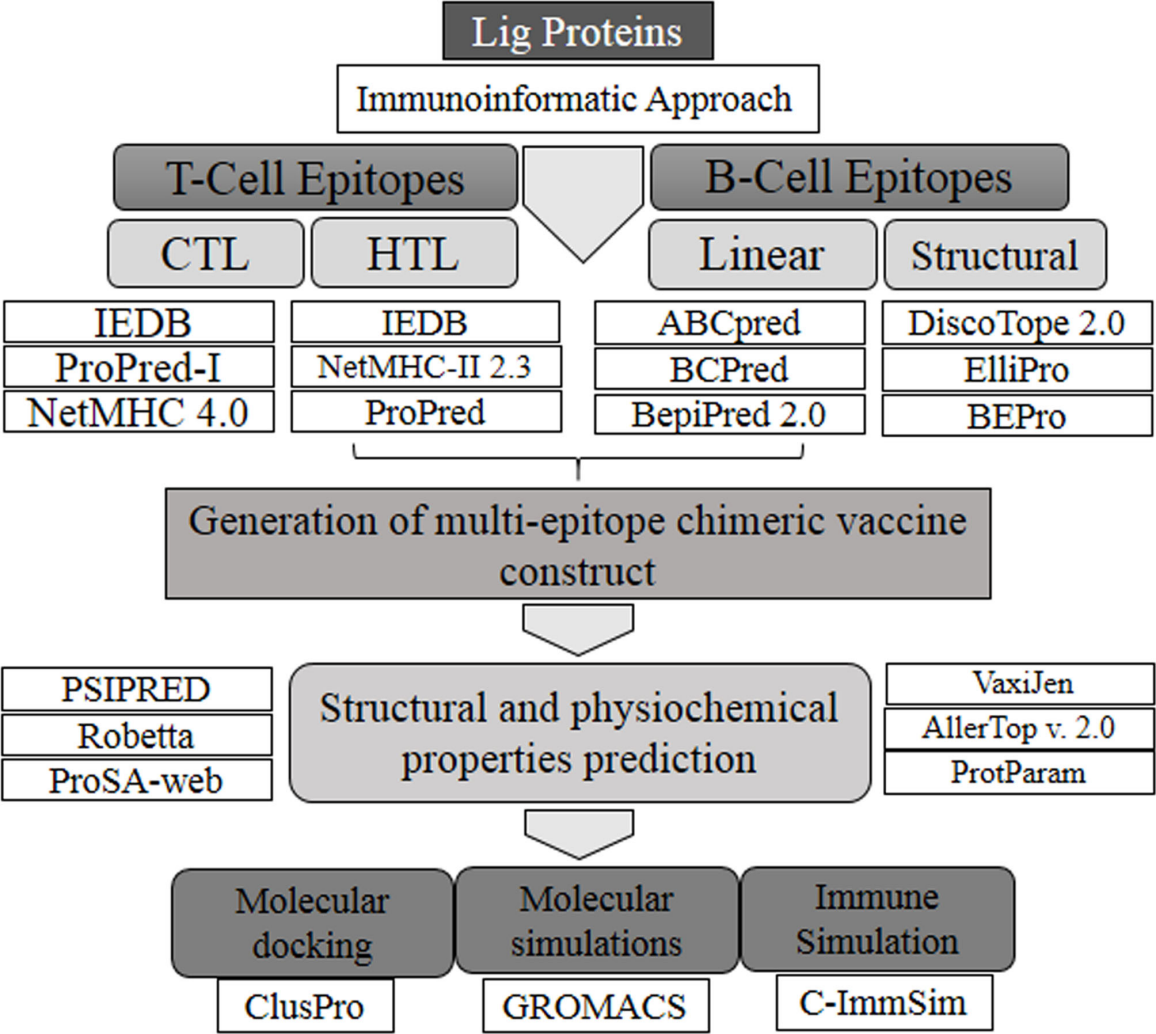
Flowchart of the methodology followed for this study.

The sequences corresponding to individual Ig-like domains were separated from LigA and LigB. LigA consists of 1,224 residues that form 13 Ig-like domains. On the other hand, LigB possesses 1,890 residues that are divided into 12 Ig-like domains and 766 residues as non-random C-terminal extensions. Each Ig-like domain consists of approximately 90 residues and is connected to the adjacent Ig-like domains by three amino acid linkers. Since the first six and half domains are conserved among the LigA and LigB proteins, the sequences corresponding to all 12 Ig-like domains from constant and variable regions of LigB (without non-repetitive C-terminal domains) were considered for the analysis, while the sequences corresponding to only variable Ig-like domains of LigA were used for antigenic epitope identification. The methods used for epitope prediction are shown in **Figure 1**.

### Identification of Linear and Conformational BCL Epitopes

In order to identify B-cell epitopes, we generated a 3D model of all individual Ig-like domains from both proteins. Good *Z*-scores and fairly good Ramachandran statistics verify the quality of the 3D models of the individual domains (**Supplementary Table S1**). We used three different servers—DiscoTope, Ellipro, and BEpro—to identify conformational (non-continuous) B-cell epitopes. All the predicted epitopes, for each Ig-like domain, from the three servers were matched and only the common and overlapping epitopes for each case were chosen. The three servers yielded different numbers of discontinuous epitopes; common epitope residues were selected. In the case of LigA7, DiscoTope and BEpro predicted 13 residues that are involved in the formation of a conformational B-cell epitope, while Ellipro, for the same case, yielded three epitopes. One among the three was observed to be common and overlapping with the former. The common selected epitope also showed a good antigenic score (**Supplementary Table S2**). A similar approach was followed to select the overlapping or partial overlapping epitopes from the other Ig-like domains of Lig proteins. The number of epitopes and residues involved and their antigenic scores predicted for each Ig-like domain are listed in **Supplementary Table S2**. The bold residues in the list depict the common and overlapping epitopes. Prediction with different servers increases the confidence of selection of epitopes.

Similarly, three servers, namely, ABCPred, BCPred, and BepiPred 2.0, were utilized for the identification of linear BCL epitopes. A total of 97 epitopes were predicted by ABCPred. LigA8 shared maximum linear BCL epitopes. BCPred predicted a total of 68 linear epitopes, and the LigA8, LigB4, and LigB6 Ig-like domains possessed significantly high numbers of epitopes. The Bepipred 2.0 server generated a total of 71 epitopes, and LigA11 was observed to have maximum epitopes. For each Ig-like domain, the epitopes predicted by all three servers were matched and the common and overlapping ones were sorted out. The linear epitopes for each Ig-like domain generated by each server and their positions are listed in **Supplementary Table S3**. The final BCL epitopes were selected based on their presence, as completely or partially, in both linear and conformational predicted epitopes. This selection criterion yielded five such epitopes from each Lig protein. The presence of these epitopes on the Ig-like domains and their length and location are listed in **Table 1**.

**Table 1:** Selected common epitopes.

Domain	MHC-I	MHC-II	B-cell epitopes	Selected region	Score
LigA9	ATISNTKGY (47–55)	SNNSVATISNTKG (42–54)	NTKGYQGQAHGTGT (51–64)	ATISNTKGYQGQAHGTGT (47–64)	1.779
LigA7	VEIQITPAA (3–11)	VEIQITPAA (3–11)	IQITPAAASKAKGLT (5–19)	VEIQITPAAASKAKGLT (3–19)	0.950
LigA7	GTVKVTASM (65–73)	TVKVTASMGG (66–75)	LGSTLKQGTVKVTA (60–71, 76, 77)	LGSTLKQGTVKVTASMGG (60–77)	0.879
LigA13	TISLSSISK (6–14)	IVNITISLS (2–9)	SSISKTKGSTHQFK (10–24)	TISLSSISKTKGSTHQFK (6–24)	0.844
LigA11	EVIPNNISF (4–12)	VIPNNISFA (5–13)	VIPNNISFAKGNSYQFKATG (5–24)	VIPNNISFAKGNSYQFKATG (5–24)	0.648
LigB12	TVSKQFFAV (15–23)	ISPINTNINTTV (5–17)	ISPINTNINTTVS (5–17)	ISPINTNINTTVSKQFFAV (5–23)	0.740
LigB12	KQFFAVGTY (18–25)	FFAVGTYSA (20–27)	FFAVGTYSAGTKAD (20–33)	KQFFAVGTYSAGTKAD (18–33)	0.694
LigB8	MVNNVTGSV (48–56)	MVNNVTGSV (48–56)	VNNVTGSVTTVA (49–60)	MVNNVTGSVTTVA (48–60)	0.648
LigB9	TSIEITPTI (1–9)	SIEITPTINS (2–10)	PTINSITHGLTKQF (7–20)	TSIEITPTINSITHGLTKQF (1–20)	0.563
LigB1	IKAEYNGLY (65–73)	IQGNRVRGI (52–60)	RVRGIASGSSIIKAEYNGLYSEQKITV (56–82)	IQGNRVRGIASGSSIIKAEYNGLYSEQKITV (52–82)	0.433

Score represents antigenicity score of the selected peptides.

### Identification of CTL and HTL Epitopes

CTL epitopes were identified among the Lig proteins using three different servers: IEDB, ProPred-1, and NetMHC. The IEDB server generated 400 strong MHC-1 binding epitopes covering 35 HLA alleles for LigA and 36 HLA alleles for LigB, and maximum number of epitopes were observed to bind with the HLA-A*68:02 allele from both proteins. LigA contributed 27 peptides and LigB shared 52 peptides toward binding to the HLA-A*68:02 allele. ProPred-I generated 1,032 epitopes that cover all 47 HLA alleles and showed maximum binding with the HLA-B*58:01 allele. LigA and LigB contributed 176 and 112 peptides toward binding to this allele, respectively. NetMHC 4.0 generated 327 epitopes that cover 72 alleles for LigA and 76 alleles for LigB. NetMHC 4.0 predicted the maximum number of peptides for the HLA-A*11:01 allele in the case of LigA (18 peptides) and HLA-C*15:02 in the case of LigB (26 peptides) (**Supplementary Table S4**). Epitopes that were overlapping or partially overlapping in at least two out of three servers and showed promiscuous nature, i.e., binding to three or more HLA alleles, were further selected for vaccine design.

In the case of HTL epitopes, the IEDB server predicted a total of 185 strong MHC-II binding epitopes (maximum binding to HLA-DRB1*07:01 for LigB and equally to HLA-DRB1*04:23, HLA-DRB1*04:26, and HLA-DRB1*04:10 for LigA), the ProPred server predicted 177 epitopes (the DRB1_0402 allele produced maximum epitopes for LigB and the DRB1_0423 for LigA), and the NetMHCII 2.3 server predicted 665 epitopes (HLA-DQA10201–DQB10303 generated maximum binding for both LigA and LigB) (**Supplementary Table S5**). After considering the promiscuous HTL epitopes, the numbers of epitopes for each case were sorted as 24, 129, and 215 from the IEDB, ProPred, and NetMHCII2.3 servers, respectively. The best candidates from each server were selected based on the IFN-γ-positive epitopes. Finally, the HLA epitopes showing their presence as either completely or partially overlapping in at least two out of three servers were considered for the vaccine design. The final selected epitopes were able to map only on four Ig-like domains of each Lig protein (**Table 1**).

### Generation of the Multi-Epitope Chimeric Vaccine

**Figure 2:**
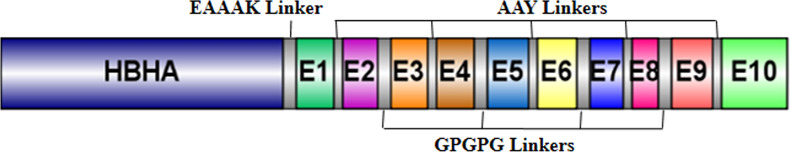
Schematic diagram of the multi-epitope vaccine construct.

The five best CTL, HTL, and BCL epitopes from LigA and LigB were used to construct multi-epitope vaccines. The selected epitopes predominantly present on Ig-like domains were as follows: LigA7, LigA9, LigA11, and LigA13 of the LigA protein and LigB1, LigB8, LigB9, and LigB12 of the LigB protein. The antigenic scores of these epitopes varied from 0.4 to 1.77 (**Table 1**). These epitopes were linked by short amino acid linkers such as AAY and GPGPG. To enhance the possibility of inducing a good immune response, the chimeric vaccine was additionally fused with a well-known TLR4 agonist, mycobacterial HBHA, by the EAAAK linker. The best combination of these epitopes was selected based on its stability and solubility. The final chimeric vaccine construct was 360 amino acids long (**Figure 2**) and was devoid of any sequence homology with the human protein sequences.

### Physicochemical Features of the Chimeric Vaccine Construct

The predicted solubility score of the vaccine construct was observed as −0.007, which suggests that it is soluble upon overexpression in a heterologous expression system. The stability index was observed to be 13.99, which categorized it as a stable protein molecule. The overall molecular weight was calculated to be 36.6 kDa. The estimated half-life in mammalian reticulocytes (*in vitro*) was 30 h, >20 h in yeast (*in vivo*), and was >10 h in *E. coli* (*in vivo*). Finally, the antigenicity and allergenicity scores of the vaccine classified it as a non-allergen for humans and a probable antigen, which can induce an immune response when used as a vaccine. All the predicted features of the vaccine construct are summarized in **Table 2**.

**Table 2:** Physicochemical features of the chimeric vaccine construct.

Physicochemical properties	
Molecular weight (kDa)	36.6
Instability index	13.99 (stable)
GRAVY score	−0.007 (soluble)
Estimated half-life	30 h (mammalian reticulocytes, *in vitro*)
	>20 h (yeast, *in vivo*)
	>10 h (*E. coli*, *in vivo*)
Ext. coefficient	14,900
Allergenicity (AllerTop 2.0)	Non-allergen
Antigenicity score (VaxiJen 2.0)	0.6848 (antigen)

GRAVY, grand average of hydropathicity index.

### Secondary and Tertiary Structure Prediction

**Figure 3:**
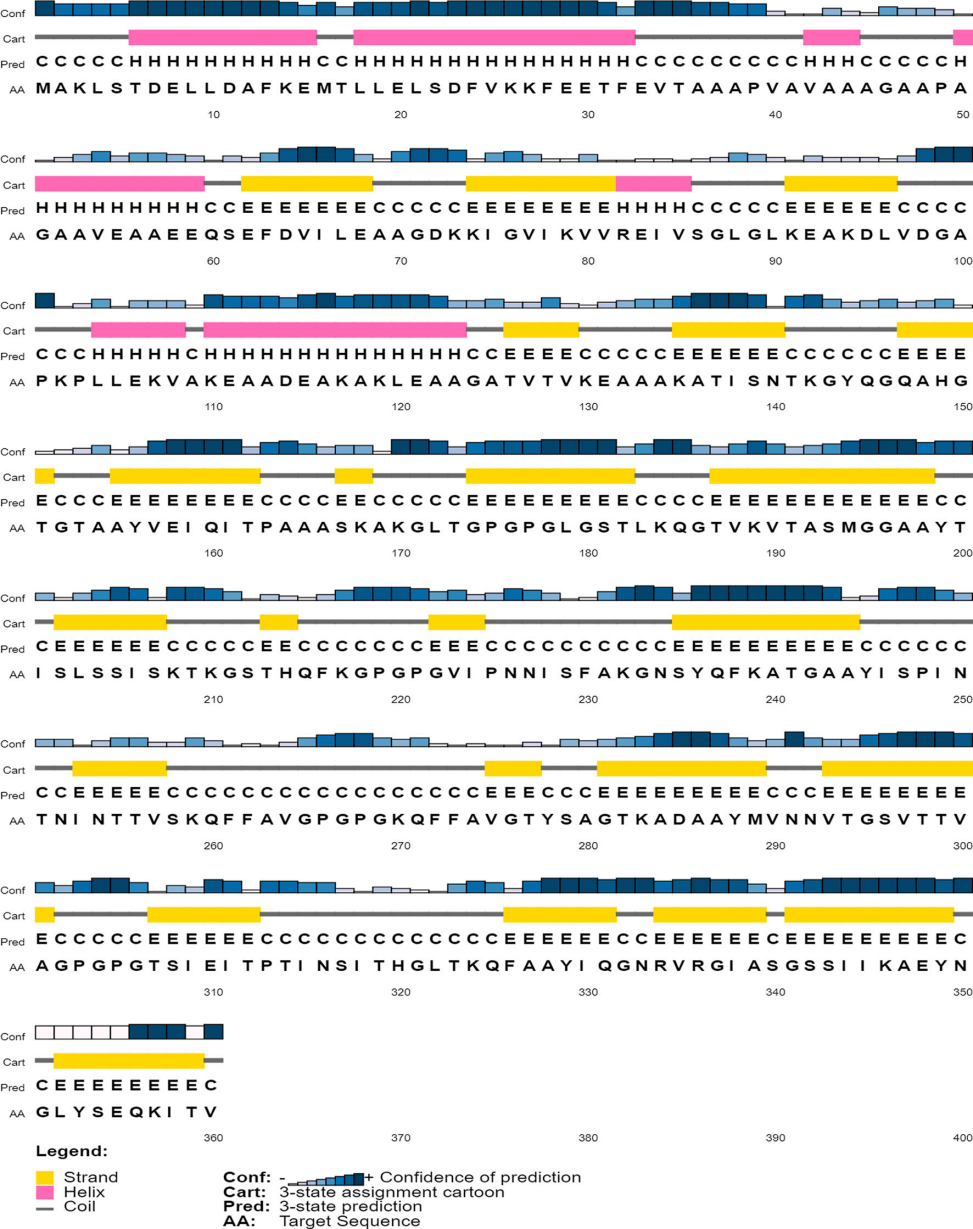
Secondary structure content of the vaccine construct.

**Figure 4:**
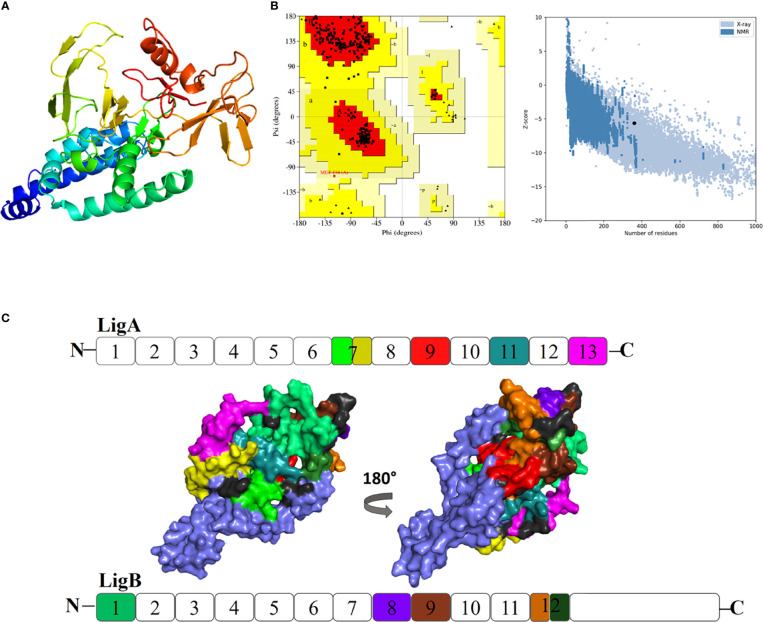
**(A)** Three-dimensional (3D) model of the vaccine construct. **(B)** Plot showing validation of the 3D model using Ramachandran plot and the ProSA server. **(C)** Promiscuous epitopes used in the vaccine construct are mapped with the *same colour code* on the Ig-like domains of Lig proteins, LigA and LigB. The N and C represent as amino and carboxy termini of the protein, respectively.

The secondary structure content of the vaccine construct included 16.96% α-helix, 41.38% extended strand, and 41.66% random coil. The residues and region of the vaccine construct involved in the formation of the secondary structure are highlighted in **Figure 3**. The 3D model of the chimeric vaccine construct showed fairly good Ramachandran plot statistics (**Figure 4A**). Approximately 99.7% of the residues were observed in the favored and additionally allowed region, and only 0.3% of residues were present in the generously allowed regions of the Ramachandran plot (**Figure 4B**). These statistics suggested that the 3D model possessed fairly ideal bond lengths and bond angles. The calculated *Z*-score of 5.61 from the ProSA server also validated the 3D model of the vaccine construct. The Ramachandran and *Z*-score plots of the same are shown in **Figure 4B**. The 3D model of the final vaccine construct consisting of epitope regions from different Ig-like domains is shown in **Figure 4C**.

### Docking Complex of the Final Vaccine Construct With Immune Receptor

**Figure 5:**
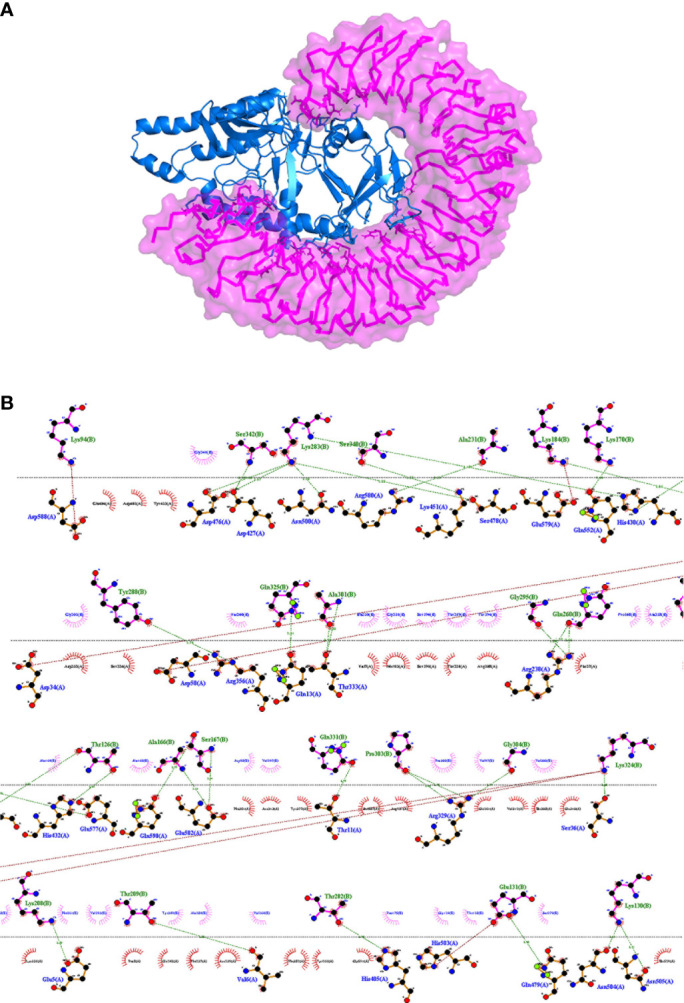
**(A)** Representation of a docked complex of the vaccine construct and TLR4 shown as a *marine blue cartoon* and a *magenta surface*, respectively. **(B)** Two-dimensional representation of the interactions between the vaccine construct and TLR4. *Red dotted lines* represent salt bridges and *green dotted lines* represent hydrogen bonds. The residue in TLR4 PDB is renumbered from 1. The start residue 27 in TLR4 PDB is represented as 1.

In order to obtain insights into the binding of the final vaccine construct with the relative immune receptors, a molecular docking study was executed with TLR4. The molecular docking analysis generated more than 30 different clusters. Cluster 1 showed the highest binding score and thus considered for further analysis. The interacting residues between the two were analyzed using LIGPLOT v2.2, which showed a strong interaction involving 32 hydrogen bonds and five salt bridges (**Table 3**). In the 3D model of the docked complex, the vaccine construct was observed to interact at the concave side of TLR4 (**Figure 5A**). The residues from the both are involved in polar interaction and salt bridges, are represented in a 2D plot (**Figure 5B**).

**Table 3:** List of residues involved in forming polar interaction and salt bridges.

Interactions	Receptor residues	Ligand residues
Hydrogen bonds	GLU-5, VAL-6, THR-11, GLN-13, SER-36, ARG-238, ARG-329, THR-333, ARG-356, HIS-405, ASP-427, HIS-430, HIS-432, LYS-451, ASP-476, SER-478, GLN-479, ASN-500, ASN-504, GLN-505, GLN-552, GLU-577, ARG-580, GLU-582, GLN-590	LYS-208, THR-209, GLN-331, GLN-325, LYS-324, GLN-260, GLY-295, PRO-303, GLY-304, ALA-301, TYR-288, THR-282 SER-342, THR-126, LYS-283, SER-340, GLU-131, LYS-130, LYS-170, LYS-184, ALA-231, ALA-166, SER-167
Salt bridges	GLU-5, ASP-34, ASP-58, ASP-476, HIS-503, GLU-577, GLU-579, ASP-588	LYS-208, LYS-324, LYS-324, LYS-283, GLU-131, LYS-184, LYS-184, LYS-94

The residues from the receptor, TLR4, are renumbered from 1. The start residue 27 in TLR4 is represented as 1.

### Molecular Dynamics Simulation

**Figure 6:**
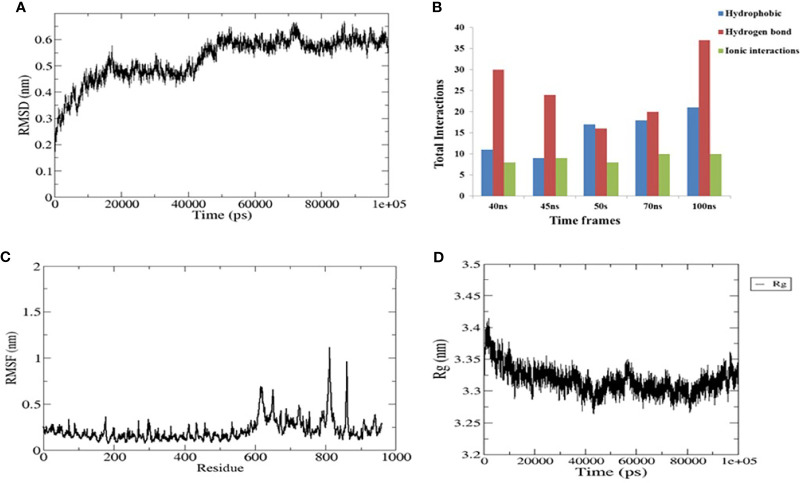
Plots of the molecular dynamics simulation (MDS) of the vaccine construct and the TLR4 complex. **(A)** Backbone root mean square deviations (RMSD). **(B)** Interface interaction plot between the construct and TLR4. **(C)** Residue-wise root mean square fluctuation (RMSF) plot. **(D)** Radius of gyration. The start residue 27 in TLR4 PDB is represented as 1.

To evaluate the stable interactions and dynamic behavior of the multi-epitope vaccine construct with TLR4, MD simulation was performed for the docked complex at 100 ns. The stability of the complex was investigated using some important parameters. The RMSD trajectory of the complex represents the structural variations associated within the overall structure of the complex formation. It indicates that the system has attained adequate stability after 50 ns of the MD simulation (**Figure 6A**). The RMSD trajectory of each protein in the complex was analyzed (**Supplementary Figure S1**). The RMSD of the complex started with 0.2 nm and reached 0.65 nm after stabilization; this was maintained until the end of the simulations, which suggests that the structural arrangement for the formation of the complex was stable. However, the RMSD plot reflected significant structural deviations at the 40-, 45-, and 70-ns time points. Therefore, to validate the convergence after 50 ns, we compared the change in interface interactions at each deviated time point along with the stable time frames. Although there was a slight deviation in the backbone, the overall interactions were maintained until the end of the simulations (**Figure 6B**).

The RMSF of all residues were computed for the docked complex. The RMSF trajectory highlights the flexibility of the residue in the docked complex. From our results, residues 0–600 showed relatively low RMSF, which indicates that these residues may be involved in certain interactions to stabilize that region (**Figure 6C**). In contrast, the construct residues 800–820 were found to have elevated RMSF values, indicating a slightly higher flexibility in these regions. Additionally, we performed a separate 50-ns MD simulation for only the construct part in order to check its flexibility. It was observed that the residual RMSF also followed the same trend as that in the complex and did not exceed more than 1.1 nm (**Supplementary Figure S2**).

The compactness of the complex was evaluated with the change in the radius of gyration (*R*_g_). It was observed that, at the start of simulations, the complex had higher *R*_g_ values, but it attained a steady *R*_g_ trend after 50 ns (**Figure 6D**). This fluctuation in *R*_g_ might be due to the folding and unfolding nature of the protein. For precision, we analyzed the separate *R*_g_ trajectories for TLR4 and the construct, which clearly indicated that both proteins have attained a flat trajectory, suggesting the compactness of the complex (**Supplementary Figure S3**).

### Immune Response Profile

**Figure 7:**
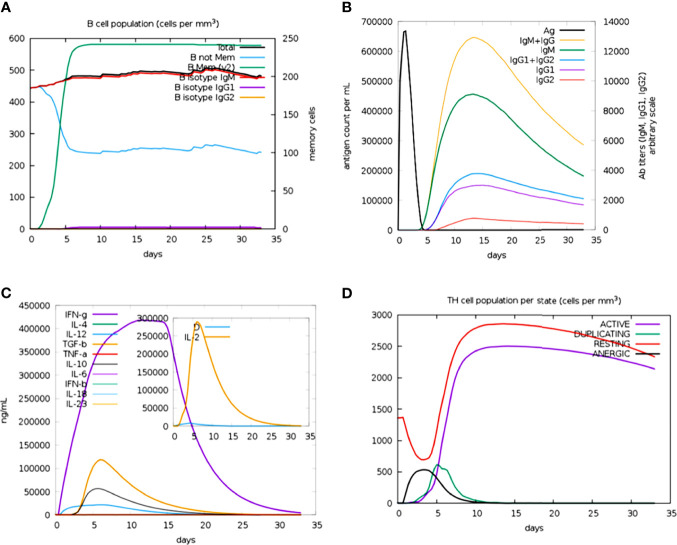
Prediction of immune response against the generated chimeric construct. **(A)** Total B-cell responses. **(B)** Total antibody responses. **(C)** Interleukin responses. **(D)** Total T-cell responses.

The immune profile of the designed vaccine construct was analyzed using an *in silico* immune simulation approach. The results of the immune simulation revealed the consistency of the actual immune response of the vaccine construct. A high level of B-cell population was observed upon administration of the vaccine construct (**Figure 7A**). At the same time, higher titration values of IgM+IgG and IgM were recorded after 15 days (**Figure 7B**). This indicates the induction of secondary and tertiary immune responses. A rise in the level of IFN-γ was observed after 5 days of incubation (**Figure 7C**). The constructed vaccine showed an increase in the population of T helper cells, thus indicating the activation of a cell-mediated immune response (**Figure 7D**). This analysis ensures the clearance of antigen by the induction of immune responses.

### *In Silico* Cloning

**Figure 8:**
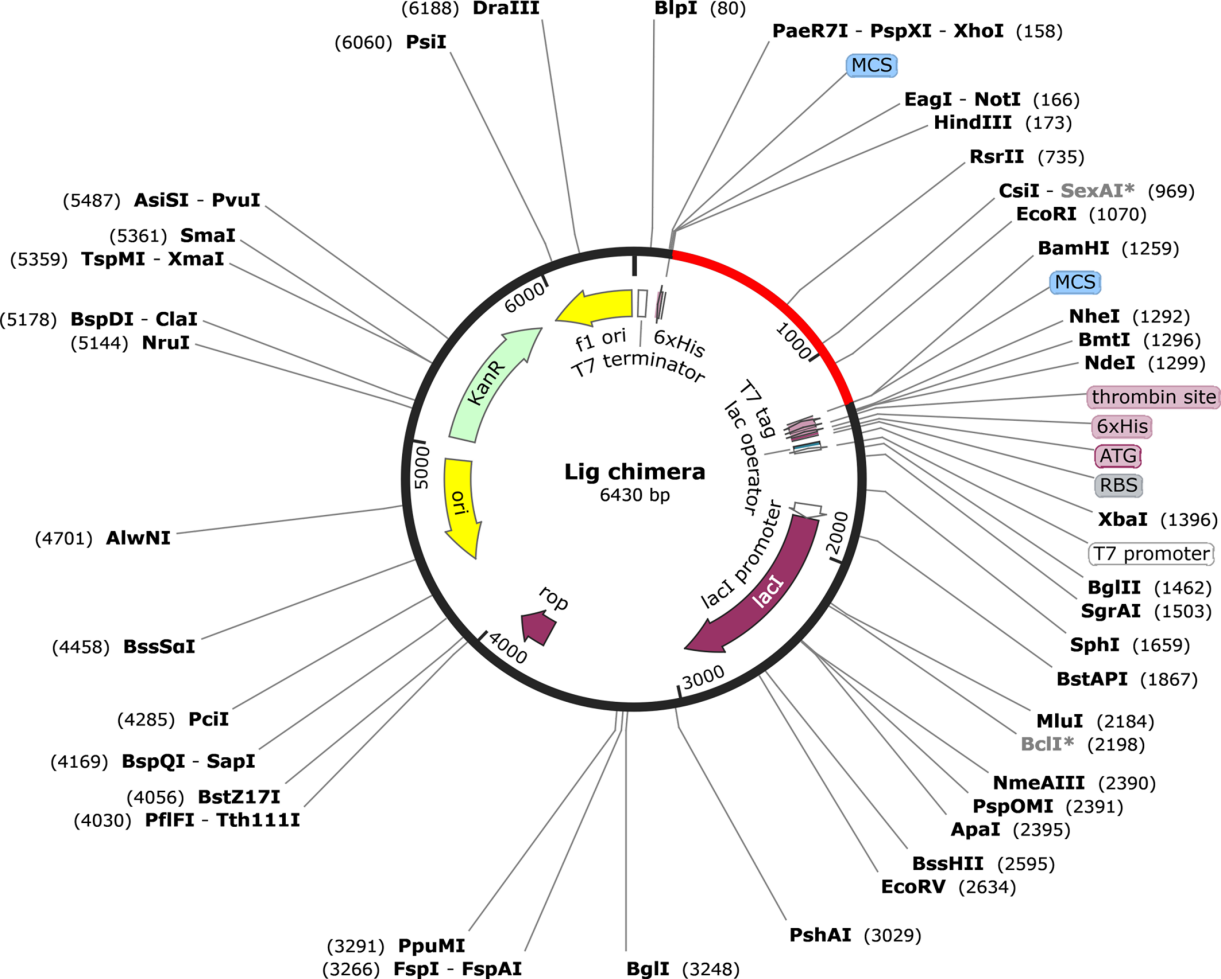
Representation of the *in silico* cloning of the multi-epitope vaccine construct. The codon optimized gene sequence of the vaccine candidate (represented in red color) was cloned between the BamHI and HindIII restriction sites of the pET-28a(+) expression vector (shown as black circle).

The codon adaptation index (CAI) value of the optimized DNA sequence was calculated to be 1, which is an ideal value and increases the chance of expression in *E. coli*. The GC content was also in the optimal range (49.62%). The expression vector pET28a(+) carrying the multi-epitope vaccine is shown in **Figure 8**. The final clone consisted of a total of 6,430 bp of DNA including the vaccine sequence (1,080 bp).

## Discussion

Obtaining accurate knowledge of antigenic epitopes from several big or multi-domain proteins has been a laborious task due to constraints associated with the lack of high expression and purification and the subsequent structural studies. Moreover, immunological research works in the context of vaccine design are experimentally costly and very intensive. However, the development of various powerful computational tools in the last one or two decades has made tremendous progress toward handling larger chunks of genomics and proteomics data for useful purposes. One such example of reverse vaccinology and structural bioinformatics has been exploited successfully to reduce the effort of screening several antigens and immuno-dominant epitopes toward the aim of designing effective vaccines. Similarly, immunoinformatics approaches have been used to unveil crucial immune-dominant determinants from available databases to design vaccines.

We have used an immunoinformatics approach to construct a multi-epitope chimeric vaccine from multi-domain outer surface proteins, *Leptospira* immunoglobulin-like (Lig) proteins. Currently, there is no effective vaccine against the global zoonotic disease leptospirosis, which accounts for approx. 60,000 human lives lost annually (1). Outer membrane proteins (OMPs) from *Leptospira* play key roles in establishing infection and are reported to be involved in immune evasion strategies (64). Many of these are being investigated for their potency as vaccine candidates. To date, the Lig family of proteins (LigA and LigB) has been reported to be the most potent vaccine candidate against leptospirosis (12, 76, 77). Humoral and cell-mediated immunities are triggered during *Leptospira* infection. Humoral immunity is majorly involved in the clearance of *Leptospira* through the production of antibodies such as IgG and IgM against leptospiral LPS and other antigenic proteins (65, 66). In fact, many other reports demonstrated the implication of cell-mediated immune responses also against leptospirosis. One such is that by Guo et al., where they demonstrated the identification of Lig peptides associated with human CD8^+^ T lymphocytes (67). Similar studies also illustrated the induction of CD4^+^ and γ/δ T cells to stimulate type 1 cytokine production against heat-killed *Leptospira* (68, 69). The potential immunoprotection of Lig proteins and their truncated version in the context of vaccine candidate and their role in T-cell- and B-cell-mediated immune response against *Leptospira* have been highlighted (17).

Our study predicted most antigenic BCL and CTL epitopes present on both LigA and LigB proteins by using multiple tools. The advantage of using multiple tools was having greater confidence in the prediction of potential epitopes. The selected CTL epitopes had strong binding with the respective HLA alleles that cover the wide distribution among the global population. Moreover, promiscuous peptides were selected, followed by separating non-self and IFN-γ-inducing epitopes. Selecting non-self-peptides may help avoid any deleterious autoimmune responses. The selection of IFN-γ epitopes is believed to induce innate and adaptive immunity (70). B-cell epitopes present in a vaccine construct help in the induction of cell-mediated immunity and contribute as an antibody-based immune therapeutics (71, 72). The generation of sequence-based linear and structure-based conformational BCL epitopes in our study using multiple tools helped us to select the best overlapping potential epitopes. Interestingly, comparison of the BCL and CTL epitopes further helped in sorting out those common and overlapping among them, yielding the final peptides for vaccine design. These peptides were mapped on the Ig-like domains of Lig proteins and observed to be present mainly in 7, 9, 11, and13 Ig-like domains from LigA and in 1, 8, 9, and 12 Ig-like domains from LigB (**Table 1**). Interestingly, it was observed that most Ig-like domains from the C-terminal portion of Lig proteins possessed high antigenic epitopes. In the case of LigA, a previous study reported that its carboxyl terminal region provides protective immunity against *Leptospira* infection (11). Moreover, Continho et al. screened within the carboxyl terminal of LigA and reported that an immunoprotective effect is mainly present in 10–13 Ig-like domains of LigA (73). No protective effect was reported from the N-terminal region of Lig proteins. In another study, the LigA variable region incorporated with poly(lactic-*co*-glycolic acid) (PLGA) also conferred protective immune response (74). Similarly, the involvement of LigB and its variant toward inducing protective immune response against *Leptospira* infection has already been highlighted (14, 75) Our prediction also showed the presence of the highest antigenic epitopes from the variable region of Lig proteins, which is in line with the previously reported studies, except for the presence of antigenic epitopes on the first Ig-like domain from the constant region of LigB. The chimeric vaccine construct generated from the selected epitopes may provide good immune response.

Our final construct consisted of the highest antigenic epitopes from these two proteins linked together by AAY and GPGPG linkers. The linkers AAY and GPGPG used for the vaccine construct have been reported to have better epitope presentation ability and to provide better sites for proteasomal system cleavage (44, 46, 78). The adjuvants used in the vaccine construct were known to induce robust immune response *via* an immune receptor. Our vaccine construct was also augmented with the adjuvant HBHA at the N-terminal. This novel adjuvant is a TLR4 agonist expressed by *Mycobacterium* known to induce a Th1-type response that helps in effective immunotherapeutic strategies (79, 80). We believe that the final construct reported in our study consists of all the required components for a vaccine construct that may also provide a better immune response (**Figure 2**).

The success of any vaccine construct relies on the fact that it should not confer any deleterious effect in the host. Our vaccine construct passes the test in this regard and has been categorized as non-allergic and antigenic, which may provide the required response. Moreover, it is predicted to be stable on overexpression and thus qualifies under a good vaccine category.

Many promising vaccine constructs follow a well-defined immunological mechanism to trigger the expected response in the host. These responses mainly lie in the successful interaction of the vaccine constructs with Toll-like receptors. In the context of *Leptospira*, it is reported that mice defective in TLR4 have increased susceptibility to *Leptospira* infection (81). One of the surface adhesins, Lsa21 from *Leptospira*, is known to induce TLR4-mediated inflammatory response (82). These studies signify the importance of host TLR4 in the immunological mechanism against leptospiral antigens. Here, we observed that our multi-epitope vaccine construct also established a protein–protein docking interaction with TLR4 through several hydrogen bonds and salt bridges (**Figure 4** and **Table 3**). The RMSD generated in the MD simulations emphasized the stable interaction between our vaccine construct and the TLR4 receptor. Moreover, the interaction and compactness of the complex between the two were also indicated in the RMSF and *R*_g_ plots (**Figure 6**). This suggests that our vaccine construct may follow a TLR4-mediated immune response.

The ultimate aim of any vaccine is to produce a good immune response in terms of antibody and cytokine productions. Hence, immune profiling of the vaccine construct is important. Our vaccine construct showed an increase in IgM antibodies after 5 days of vaccination, a rise in the B-cell population, and elevated levels of IFN-γ. The predicted immune profiles support the strong cellular and humoral responses associated with our vaccine construct. Finally, the production of a recombinant vaccine construct requires a suitable heterologous system for expression. Since the half-life of our vaccine construct is predicted to be fairly stable (>10 h) in the *E. coli* expression system, the *E. coli* strain can be used as a source for heterologous expression. Based on the *E. coli* strain K12, codon optimization of the vaccine construct gene was performed for the *in silico* cloning of the same in a common expression vector, pET28a(+).

## Conclusion

Multi-domain outer surface proteins, such as LigA and LigB, have been reported to be vaccine candidates. These two consist of several (12–13) Ig-like domains with an overall size of proteins ranging from approximately 110 to 220 kDa. It has always been challenging to express and purify these proteins for immunological studies. Our immunoinformatics studies have exploited multiple tools to identify the best antigenic epitopes between the two proteins. The common and overlapping epitopes generated from these multiple tools were considered for the design of a multi-epitope chimeric vaccine construct. The use of multi-epitopes has provided confidence in epitope selection. We believe that our multi-epitope chimeric vaccine construct might provide better results both for *in vitro* and *in vivo* assays.

## Data Availability Statement

The original contributions presented in the study are included in the article/**Supplementary Material**. Further inquiries can be directed to the corresponding author.

## Author Contributions

PK and MA conceived the idea and designed the experiment. PK performed most of the analysis and wrote the paper. SL performed and analyzed the MD simulation and helped in writing the molecular simulation part. US helped in the docking. MA supervised the study and edited the article. All authors contributed to the article and approved the submitted version.

## Funding

The research work is supported by SERB grant number “EMR/2016/001183” of Govt. of India. We thank for the partial financial supports from UoH-IoE by MHRD (Sanction no. UoH/IoE/RC01/RC1-20-017)) as well as UGC-SAP-DRS-1 to the Department of Biochemistry, School of Life Sciences, University of Hyderabad (UoH).

## Conflict of Interest

The authors declare that the research was conducted in the absence of any commercial or financial relationships that could be construed as a potential conflict of interest.

## Publisher’s Note

All claims expressed in this article are solely those of the authors and do not necessarily represent those of their affiliated organizations, or those of the publisher, the editors and the reviewers. Any product that may be evaluated in this article, or claim that may be made by its manufacturer, is not guaranteed or endorsed by the publisher.
