# Supplementary material for: Immunoinformatics-Based Designing of a Multi-Epitope Chimeric Vaccine From Multi-Domain Outer Surface Antigens of Leptospira
Source: Front Immunol. 2021 Nov 30;12:735373. doi: 10.3389/fimmu.2021.735373 (PMC8670241; doi:10.3389/fimmu.2021.735373)
Supplement: Supplementary file 1 [file DataSheet_1.pdf]

Supplementary information

Immunoinformatics based designing of a multi-epitope chimeric vaccine from multi-domain outer surface antigens of Leptospira

Pankaj Kumar[#], Surabhi Lata[#], Umate Nachiket Shankar, Mohd. Akif*

Laboratory of Structural Biology, Department of Biochemistry, School of Life Sciences, University of Hyderabad, Hyderabad, (500046), INDIA

# Authors contributed equally

*Correspondence: Mohd. Akif,

Email: akif@uohyd.ac.in

Table S1: Ramachandran statistics and ProSA Z score

Protein	Favoured Region (%)	Allowed Region (%)	Outlier Region (%)	ProSA Z-Score
LigA7	94.3	5.7	0	-3.64
LigA8	92.7	6.1	1.2	-4.47
LigA9	93	7	0	-4.47
LigA10	95.3	3.5	1.2	-4.18
LigA11	94.1	5.9	0	-4.66
LigA12	92	6.9	1.1	-5.17
LigA13	92	6.9	1.1	-4.35
LigB1	95.3	4.7	0	-4.96
LigB2	96.4	2.4	1.2	-3.88
LigB3	96.3	3.7	0	-4.01
LigB4	96.5	2.3	1.2	-4.10
LigB5	97.6	1.2	1.2	-3.50
LigB6	97.7	1.1	1.1	-3.82
LigB7	94.3	4.5	1.1	-2.98
LigB8	94	6	0	-3.10
LigB9	94.3	4.5	1.1	-3.73
LigB10	94.3	4.5	1.1	-3.17
LigB11	95.3	3.5	1.2	-2.77
LigB12	95.5	3.4	1.1	-4.70

Table S2: List of predicted conformation BCL epitopes

Domain	BEPro	DiscoTope 2.0		ElliPro		
	Residue No.	Residue No	Total	Residue	Total	Score
LigA7	1-2, **30-33,36,** 54, 56-59	2, 5, **29-36**, 56, 58, 59	13	A:A1, A:K2, A:L3, A:V4, A:E5, **A:F29, A:T30, A:D31, A:N32, A:S33, A:N34,** A:G78, A:G79	13	0.791
				A:N55, A:T56, A:N57, A:A58, A:K59	5	0.771
				A:A11, A:A12, A:A13, A:S14, A:K15, A:A16, A:K17, A:G18, A:S46, A:N47, A:T48, A:D49, A:I50, A:L66, A:K67, A:Q68, A:G69, A:T70, A:T87, A:T89, A:Q90	21	0.67
LigA8	1, 12, **24-32**, 51-54, 74	1, 22, **24-32**, 36, 52-54,	15	A:S1, **A:I24, A:F25, A:T26, A:D27, A:H28, A:S29, A:K30**	8	0.833
LigA8	1, 12, **24-32**, 51-54, 74	1, 22, **24-32**, 36, 52-54,	15	A:I33, A:A51, A:P52, A:G53, A:E54, A:E55	6	0.72
				A:T34, A:E35, A:Q36, A:E73, A:K74	5	0.652
				A:T7, A:A9, A:S10, A:A12, A:K13, A:G14, A:M15, A:S42, A:S43, A:K44, A:A45, A:L46, A:I61, A:A62, A:V63, A:G64, A:N65, A:T82, A:T84	19	0.647
LigA9	1, **26-31**, 51, 53-56	2, 4, **23, 25, 27-33**, 54, 55,	13	A:T1, A:S2, A:I25, **A:Y26, A:S27, A:D28, A:N29, A:S30, A:S31,** A:K32, A:I34, A:T35, A:S36, A:S51, A:N52, A:T53, A:K54, A:G55, A:Y56, A:G75, A:N76	21	0.725
LigA9	1, **26-31**, 51, 53-56	2, 4, **23, 25, 27-33**, 54, 55,	13	A:H10, A:C11, A:L12, A:V13, A:K14, A:G15, A:S43, A:N44, A:N45, A:S46, A:V47, A:G64, A:T65, A:G66, A:T67, A:S85, A:T87, A:A88	18	0.705
				A:V8, A:K9, A:K83	3	0.561
LigA10	13, **27-30,** 54-56	13, **28, 29**, 87	4	A:A53, A:T54, A:G55, A:S56, A:K57	5	0.823
LigA10	13, **27-30,** 54-56	13, **28, 29**, 87	4	A:I1, A:E2, A:V25, **A:F26, A:T27, A:D28, A:N29, A:S30,** A:T31, A:K32, A:D33, A:I34, A:T35	13	0.804

				A:T8, A:S9, A:S10, A:H11, A:K12, A:A13, A:K14, A:G15, A:S43, A:N44, A:T45, A:A46, A:Y47, A:L63, A:S64, A:K65, A:G66, A:T67, A:Q85, A:T87	20	0.699
LigA11	**13-15,** 27-30, **47,** 55, 65,	**13-15,** 28, 29, **47,** 65, 67, 87	9	A:V1, A:S2, A:I25, A:Y26, A:T27, A:D28, A:H29, A:S30, A:E31	9	0.815
LigA11	**13-15,** 27-30, **47,** 55, 65,	**13-15,** 28, 29, **47,** 65, 67, 87	9	**A:A13, A:K14, A:G15,** A:T87	4	0.784
				A:I34, A:E51, A:T53, A:F54, A:G55, A:S56, A:A57	7	0.686
				A:N8, A:N9, A:I10, A:S11, **A:S43, A:N44, A:P45, A:K46, A:I47,** A:N64, A:I65, A:G66, A:S67, A:N85	14	0.677
				A:T35, A:E36, A:Q37, A:V38, A:S75, A:D76, A:T77	7	0.575
LigA12	1-2, 4, 6, 13, **25-33,** 53-55, **76-78,** 89	1, 2, 4, **23-33,** 35-37, 55, 73, **75-79**	24	A:R1, A:Y2, **A:T25, A:Y26, A:S27, A:D28, A:Q29, A:S30, A:T31, A:K32,** A:L34, A:T35, A:E36, A:N52, A:T53, A:P54, A:G55, A:K56, A:K57, A:Y74, **A:D75, A:H76, A:H77, A:Q79**	24	0.682
	1-2, 4, 6, 13, **25-33,** 53-55, **76-78,** 89	1, 2, 4, **23-33,** 35-37, 55, 73, **75-79**	24	A:S8, A:Y9, A:A10, A:G11, A:E13, A:K14, A:G15, A:Y16, A:S43, A:N44, A:P45, A:S46, A:S47, A:S63, A:E64, A:L65, A:G66, A:E67, A:T86, A:T88, A:E89	21	0.660
LigA13	1, 15, **29-32**, 56-57, 89	1, **29-31,**	4	A:S53, A:N54, A:V55, A:D56, A:D57, A:E58	6	0.732
	1, 15, **29-32**, 56-57, 89	1, **29-31,**	4	A:G1, A:I2, A:V3, A:N4, **A:K27, A:F28, A:E29, A:N30, A:G31, A:A32, A:E33, A:I34, A:L36, A:T37, A:E38**, A:N77, A:S78	17	0.697
				A:S10, A:S11, A:I12, A:S13, A:K14, A:T15, A:K16, A:G17, A:S45, A:N46, A:P47, A:T48, A:V49, A:L65, A:S66, A:V67, A:G68, A:S69, A:K71, A:D84, A:E86, A:T88, A:P89	23	0.646
LigB1	**1-6,** 18-19, **29-37**, 40, 49-50, 55-56, 74-76, 86-87	**1-6,** 27, **29-37,** 40, 41, 56, 74-77	23	**A:N1, A:P2, A:T3, A:T5, A:R6, A:I29, A:F30, A:D31, A:N32, A:G33, A:T34, A:N35, A:Q36**	13	0.8
LigB1	**1-6,** 18-19, **29-37**, 40, 49-50, 55-56, 74-76, 86-87	**1-6,** 27, **29-37,** 40, 41, 56, 74-77	23	A:S14, A:S15, A:I16, A:A17, A:N18, A:G19, A:S48, A:Q49, A:S50, A:V51, A:I62, A:A63, A:S64, A:G65, A:S66, A:T83, A:V84, A:T85, A:P86, A:A87	20	0.647

LigB2	**1,** 14-15, **25-32,** 38, 48, 73-75, 85-86	**1,** 14,15, **25-32,** 38, 48, 73, 74	15	**A:S1,** A:Q3, **A:I25, A:F26, A:S27, A:D28, A:G29, A:S30, A:H31**	9	0.832
LigB2	**1,** 14-15, **25-32,** 38, 48, 73-75, 85-86	**1,** 14,15, **25-32,** 38, 48, 73, 74	15	A:S9, A:G10, A:I11, A:L12, A:P13, A:K14, A:G15, A:T16, A:N17, A:S45, A:N46, A:P47, A:D48, A:L49, A:I61, A:N62, A:L63, A:G64, A:T65, A:E80, A:T82, A:V83, A:G84, A:D85, A:A86	25	0.623
				A:I34, A:S35, A:N36, A:D37, A:P38, A:L39, A:D53, A:D54, A:S55, A:G56, A:Q73, A:S74, A:K75	13	0.573
LigB3	1-2, 6-7, 9, 14-15, 17, 19, 23, **25-33,** 36, 45-46, **51-53,** 55, 71-72, 83-84	1, 2, 7, 9, 14, 15, 17, **23-33,** 36, 46, **51-53,** 59, 71, 72, 83, 84	28	A:S1, A:Q2, **A:I25, A:Y26, A:S27, A:D28, A:N29, A:S30, A:N31, A:R32,** A:I34	11	0767
LigB3	1-2, 6-7, 9, 14-15, 17, 19, 23, **25-33,** 36, 45-46, **51-53,** 55, 71-72, 83-84	1, 2, 7, 9, 14, 15, 17, **23-33,** 36, 46, **51-53,** 59, 71, 72, 83, 84	28	A:E71, A:N72, A:I73, A:I74	4	0.726
				A:N8, A:N9, A:P10, A:N11, A:I12, A:P13, A:L14, A:G15, A:K16, A:K17, A:S43, A:N44, A:S45, A:T46, A:I47, A:A59, A:D60, A:T61, A:G62, A:I63, A:I80, A:T82, A:P83, A:A84	24	0.634
				A:S35, A:S36, A:S37, **A:Q51, A:N52, A:N53**	6	0.552
LigB4	1, **26-31**, 46, **53-55,** 66, 75-76, 88	**25, 27-31**, **54, 55,** 76	9	A:V1, A:S2, **A:I25, A:F26, A:T27, A:D28, A:N29, A:S30, A:N31, A:S32, A:I34**	11	0.758
LigB4	1, **26-31**, 46, **53-55,** 66, 75-76, 88	**25, 27-31**, **54, 55,** 76	9	A:G75, A:G76, A:I77, A:Q78	4	0.716
				A:S51, A:N52, **A:A53, A:S54,** A:D55, A:S56, A:H57	7	0.695
				A:T8, A:N9, A:S10, A:T11, A:V12, A:A13, A:K14, A:G15, A:L16, A:S43, A:N44, A:T45, A:D46, A:I47, A:L63, A:N64, A:Q65, A:G66, A:N67, A:T84, A:V85, A:T86, A:Q87, A:A88	24	0.649
LigB5	1, **25-32,** 36, **52-55,** 74-75	**25-32,** **52-54,**	11	A:T1, A:S2, **A:I25, A:F26, A:T27, A:D28, A:N29, A:S30, A:K31**	9	0.854
LigB5	1, **25-32,** 36, **52-55,** 74-75	**25-32,** **52-54,**	11	A:G74, A:K75, A:V76, A:S77	4	0.713
				A:V8, A:L9, A:P10, A:S11, A:I12, A:A13, A:K14, A:G15, A:L16, A:S43, A:S44, A:A45, A:I46, A:V47, A:H62,	24	0.610

				A:A63, A:V64, A:G65, A:D66, A:T83, A:V84, A:V85, A:P86, A:A8		
				A:T35, A:D36, A:Q37, A:S48, A:V49, A:S50, A:N51, **A:L52, A:D53, A:D54,** A:N55, A:K56	12	0.573
LigB6	1-2, 7, **25-33,** 36, **53-56,** 76, 90	1, 2, **23-33,** 36, **53-55,** 76	18	A:T1, A:S2, **A:I25, A:Y26, A:S27, A:D28, A:N29, A:S30, A:N31, A:K32**	10	0.831
LigB6	1-2, 7, **25-33,** 36, **53-56,** 76, 90	1, 2, **23-33,** 36, **53-55,** 76	18	A:G75, A:K76, A:V77, A:S78	4	0.711
				A:S36, **A:S51, A:N52, A:A53, A:Q54, A:K55,** A:N56, A:Q57	8	0.697
				A:S11, A:L12, A:A13, A:K14, A:G15, A:S43, A:D44, A:S45, A:S46, A:I47, A:A63, A:A64, A:T65, A:G66, A:A67, A:V86, A:T87, A:A88, A:A89, A:K90	20	0.670
LigB7	1, **25-32,** 36, 45-46, **53-56,** 76, 89-90	**26-31,** 46, **53-55,** 90	11	A:V1, A:E2, **A:I25, A:F26, A:T27, A:D28, A:N29, A:S30, A:N31, A:S32, A:I34**	11	0.768
LigB7	1, **25-32,** 36, 45-46, **53-56,** 76, 89-90	**26-31,** 46, **53-55,** 90	11	A:A10, A:S11, A:K12, A:A13, A:K14, A:G15, A:L16, A:S43, A:N44, A:T45, A:D46, A:I47, A:E49, A:L63, A:T64, A:P65, A:G66, A:S67, A:K85, A:V86, A:T87, A:P88, A:A89, A:Q90	24	0.653
				A:T35, A:N36, A:Q37, **A:T51, A:N52, A:T53, A:S54, A:G55, A:S56, A:K57**	10	0.607
				A:L74, A:G75, A:S76, A:I77, A:K78, A:S79, A:S80	7	0.579
LigB8	**27-30,** 45-46, 52, 73, 85	**28, 29,** 46	3	A:D72, A:S73, A:I74	3	0.776
LigB8	**27-30,** 45-46, 52, 73, 85	**28, 29,** 46	3	A:I1, A:S2, **A:T25, A:Y26, A:T27, A:D28, A:H29, A:S30, A:V31, A:Q32, A:V34, A:T35, A:A36, A:L37, A:A38,** A:N51, A:N52, A:V53, A:T54, A:G55	20	0.640
				A:P7, A:I8, A:N9, A:P10, A:S11, A:V12, A:A13, A:K14, A:G15, A:L16, A:S43, A:N44, A:P45, A:R46, A:K47, A:V60, A:A61, A:T62, A:G63, A:N64, A:N81, A:V82, A:T83, A:P84, A:A85	25	0.625

LigB9	1-2, 7, 9, 14-15, **26-31,** 45-46, **51, 53-56,** 66, 75, 76, 81, **89-90**	2, 7, 9, 13-15, **26-31,** 33, 44-46, **51, 53-56,** 63, 64, 66, 67, **88-90**	28	A:T1, A:S2, **A:I25, A:F26, A:S27, A:D28, A:K29, A:S30, A:T31, A:Q32**	10	0.832
LigB9	1-2, 7, 9, 14-15, **26-31,** 45-46, **51, 53-56,** 66, 75, 76, 81, **89-90**	2, 7, 9, 13-15, **26-31,** 33, 44-46, **51, 53-56,** 63, 64, 66, 67, **88-90**	28	A:F76, A:I77, A:Q78	3	0.719
				A:T35, A:Q36, A:L37, A:E51, A:N52, **A:T53, A:S54, A:G55, A:K56, A:K57**	10	0.627
				A:T8, A:I9, A:N10, A:S11, A:I12, A:T13, A:H14, A:G15, A:L16, A:S43, A:D44, A:P45, A:S46, A:K47, A:S63, A:K64, A:L65, A:G66, A:S67, A:V86, A:T87, **A:D88, A:L89**	23	0.611
LigB10	1-2, 7, **25-31, 53-55,** 75-77, 90	1, 2, **25-31, 54, 55,** 76	12	A:K1, A:S2, **A:T25, A:F26, A:I27, A:D28, A:G29, A:S30, A:E31, A:Q32**	10	0.844
LigB10	1-2, 7, **25-31, 53-55,** 75-77, 90	1, 2, **25-31, 54, 55,** 76	12	A:N75, A:S76, A:I77	3	0.770
				A:S10, A:S11, A:I12, A:A13, A:K14, A:G15, A:L16, A:S43, A:K44, A:S45, A:D46, A:V47, A:A48, A:A62, A:L63, A:S64, A:I65, A:G66, A:S67, A:N83, A:N85, A:V86, A:S87, A:A88, A:A89, A:T90	26	0.607
				A:T35, A:N36, A:L37, A:I50, A:N51, A:N52, **A:A53, A:A54, A:N55,** A:E56, A:K57	11	0.600
LigB11	**1-2, 4,** 7, 9, 15, **25-32,** 45-46, 53-56, 75-77, 80, 87, 88	**1-4,** 6, 7, 9, **23-33,** 36, 37, 46, 53-56, 67, 73, 75-78, 80, 82	33	**A:D1, A:S2, A:K4, A:V25, A:Y26, A:S27, A:D28, A:S29, A:T30, A:I31, A:Q32**	11	0.806
LigB11	**1-2, 4,** 7, 9, 15, **25-32,** 45-46, 53-56, 75-77, 80, 87, 88	**1-4,** 6, 7, 9, **23-33,** 36, 37, 46, 53-56, 67, 73, 75-78, 80, 82	33	A:N75, A:S76, A:I77	3	0.754
				A:V8, A:N9, A:N10, A:N11, A:I12, A:A13, A:K14, A:G15, A:L16, A:S43, A:N44, A:S45, A:S46, A:S47, A:L63, A:Q64, A:I65, A:G66, A:N67, A:T84, A:V85, A:S86, A:A87, A:A88	24	0.642
				A:S35, A:D36, A:S37, A:S51, A:N52, A:S53, A:T54, A:E55, A:T56, A:K57, A:G58	11	0.544

LigB12	**14-15,** 27-30, 45-47, 53-55, 64, 66-67, 76, **86-90**	**14, 15,** 46, 63, 64, 66, 67, **86-90**	12	A:S1, A:S2, A:G24, A:T25, A:Y26, A:S27, A:A28, A:G29, A:T30, A:K31, A:A32, A:L34	12	0.749
LigB12	**14-15,** 27-30, 45-47, 53-55, 64, 66-67, 76, **86-90**	**14, 15,** 46, 63, 64, 66, 67, **86-90**	12	A:G75, A:S76, A:V77, A:S78	4	0.708
				A:P7, A:I8, A:N9, A:T10, A:N11, A:I12, A:N13, **A:T14, A:T15,** A:S43, A:N44, A:Q45, A:S46, A:Q47, A:K49, A:I63, A:A64, A:S65, A:G66, A:N67, A:T84, A:V85, **A:N86, A:K87, A:T88, A:D89, A:T90**	27	0.622
				A:T35, A:S36, A:S37, A:S51, A:N52, A:A53, A:S54, A:E55, A:T56, A:K57	10	0.614

Table S3: List of predicted linear BCL epitopes

Domain	BepiPred 2.0		BCPred			ABCPred,		
	**Peptide**	**Position**	**Peptide**	**Position**	**Score**	**Peptides**	**Position**	**Score**
LigA7	ASKAKGLT	12-19	**FTDNSNSDITNQ**	**28-39**	**0.992**	IQITPAAASKAKGL	5-18	0.87
	**FTDNSNSDI**	**28-36**	TWNSSNTDILTV	41-52	0.916	AKGLTERFKATGIF	15-28	0.87
	VSNTNAKRGLG	52-62	GLGSTLKQGTVK	60-71	0.695	QGTVKVTASMGGIE	67-80	0.80
	EDS	79-81	DSVDFTVTQATL	81-92	0.583	LGSTLKQGTVKVTA	61-74	0.80
						SNSDITNQVTWNSS	32-45	0.80
						**FKATGIFTDNSNSD**	**22-35**	**0.78**
						AAASKAKGLTERFK	10-23	0.75
LigA8	**VSPTRASIAKG**	**5-15**	**TSIEVSPTRASI**	**1-12**	**0.898**	NISITATLEKLSGK	66-79	0.86
	TDHSKKNIT	27-35	KKNITEQVTWKS	31-42	0.741	SSSKALSMLNAPGE	42-55	0.81
	KSSSKALS	41-48	LSGKTDITVTPA	76-87	0.715	**VSPTRASIAKGMTQ**	**5-18**	**0.80**
	APGEEGTGKAI	52-62	APGEEGTGKAIA	52-63	0.675	SMLNAPGEEGTGKA	48-61	0.79
			KGMTQKFTATGI	14-25	0.307	HSKKNITEQVTWKS	29-42	0.79
						GTGKAIAVGNISIT	57-70	0.75
						GIFTDHSKKNITEQ	24-37	0.75
						TQKFTATGIFTDHS	17-30	0.75
LigA9	ISPVKHCLVKGLT	5-17	TLGNVSSQVSKL	73-84	0.999	NTKGYQGQAHGTGT	52-65	0.94
	**YSDNSSKDI**	**26-34**	AHGTGTGTVDIK	60-71	0.946	**GIYSDNSSKDITSA**	**24-37**	**0.78**
	SSNN	42-45	VATISNTKGYQG	47-58	0.616	SKDITSAVTWHSSN	31-44	0.75
	NTKGYQGQ	52-59	**TGIYSDNSSKDI**	**23-34**	**0.373**			
LigA10	**NPTSSHKAKGLTE**	**6-18**	**FTDNSTKDITDQ**	**26-37**	**0.948**	**TDNSTKDITDQVTW**	**27-40**	**0.88**
	**TDNSTKDITD**	**27-36**	ISNATGSKGVVN	50-61	0.720	**LNPTSSHKAKGLTE**	**5-18**	**0.85**
	SS	42-43	SSANATFQVTPA	78-89	0.411	ITDQVTWKSSNTAY	34-47	0.84
	NATGSK	52-57	**HKAKGLTENFKA**	**11-22**	**0.338**	SHKAKGLTENFKAT	10-23	0.80
						LTENFKATGVFTDN	16-29	0.77
						KGTSHISATLGSIS	65-78	0.75

LigA11	**IPNNISFAKGNS**	**6-17**	VSTTNIGSTNIT	60-71	0.855	EADITEQVTWSSSN	31-44	0.85
	YTDHSEADI	26-34	**AKGNSYQFKATG**	**13-24**	**0.639**	FKATGIYTDHSEAD	20-33	0.85
	S	43				GSTNITAKLSDTVS	66-79	0.83
	P	45				**VIPNNISFAKGNSY**	**5-18**	**0.81**
	A	48				ASVENTFGSAGLVS	48-61	0.75
	VENTFGSA	50-57						
LigA12	YAGIEKGYT	9-17	**GTYSDQSTKDLT**	**24-35**	**0.920**	**SDQSTKDLTEDVTW**	**27-40**	**0.91**
	**YSDQSTKDLTE**	**28-36**	GEPDITVFYDHH	66-77	0.589	TKQFSAIGTYSDQS	17-30	0.82
	S	43	QSSYTPVTVTES	79-90	0.447	SNPSSVVIENTPGK	43-56	0.75
	TPGKKG	53-58				GIEKGYTKQFSAIG	11-24	0.75
LigA13	**SSISKTKGST**	**10-19**	**ISKTKGSTHQFK**	**12-23**	**0.842**	SNPTVVSISNVDDE	45-58	0.93
	ENGAEIDLT	29-37	TGKFENGAEIDL	25-36	0.585	**SSISKTKGSTHQFK**	**10-24**	**0.92**
	S	45	VVSISNVDDERG	49-60	0.585	AEIDLTELVTWSSS	32-45	0.81
	VDDERGL	55-61	SSSIDFEVTPEI	80-91	0.368	STHQFKATGKFENG	18-31	0.79
LigB1	**ELSYQDSSIANGTST**	**7-21**	DSTSIVPDSQSV	39-50	0.997	LEVTAIFDNGTNQN	23-36	0.89
	FDNGTNQNITDSTSIV PDSQS	29-49	**SIANGTSTTLEV**	**14-25**	**0.996**	ITDSTSIVPDSQSV	37-50	0.88
	**RVRGIASGSSIIKAEY NGLYSEQKITV**	**57-83**	YSEQKITVTPAT	76-87	0.613	**ELSYQDSSIANGTS**	**7-20**	**0.85**
			**TIQGNRVRGIAS**	**52-63**	**0.053**	SIIKAEYNGLYSEQ	66-79	0.80
						IANGTSTTLEVTAI	15-28	0.78
						NPTIRIELSYQDSS	1-14	0.78
						**IQGNRVRGIASGSS**	**53-66**	**0.76**
LigB2	**TSLGSGILPKGTN**	**5-17**	SSNPDLVQVDDS	45-56	0.739	**IQVTSLGSGILPKG**	**3-16**	**0.88**
	**FSDGSHQDISNDPLIV WSSSNP**	**27-48**	**SDGSHQDISNDP**	**28-39**	**0.635**	**SHQDISNDPLIVWS**	**31-44**	**0.81**
			HIRASFQSKQGA	68-79	0.556	VQVDDSGLASGINL	51-64	0.78
			**GSGILPKGTNRQ**	**9-20**	**0.553**	GINLGTAHIRASFQ	61-74	0.77
LigB3	**VTSNNPNIPLGKKQ**	**5-18**	SSNPDLVQVDDS	45-56	0.739	ILETADTGIVTISA	55-68	0.83
	**YSDNSNRDIS**	**26-35**	**SDGSHQDISNDP**	**28-39**	**0.635**	IANIQNNGILETAD	47-60	0.80
			HIRASFQSKQGA	68-79	0.556	**QIQVTSNNPNIPLG**	**2-15**	**0.78**

			**GSGILPKGTNRQ**	**9-20**	**0.553**	**LIATGIYSDNSNRD**	**20-33**	**0.77**
						PLGKKQKLIATGIY	13-26	0.75
LigB4	**SPTNSTVAKGLQ**	**6-17**	**FTDNSNSDITDQ**	**26-37**	**0.972**	**TDNSNSDITDQVTW**	**27-39**	**0.87**
	**FTDNSNSDITD**	**26-36**	TWDSSNTDILSI	39-50	0.851	NQGNVKVTASIGGI	64-77	0.81
	T	45	**ISVSPTNSTVAK**	**3-14**	**0.83**	FKATGIFTDNSNSD	20-33	0.79
	LSISNASDSHG	48-58	ASTLNQGNVKVT	60-71	0.352	**VSISVSPTNSTVAK**	**1-14**	**0.77**
			GGIQGSTDFTVT	75-86	0.316			
LigB5	SPVLPSIAKGLT	6-17	**FTDNSKKDITDQ**	**26-37**	**0.954**	SVSNLDDNKGLGKA	48-61	0.84
	**FTDNSKKDIT**	**26-35**	GDTTITATLGKV	65-76	0.944	KGLTQKFTAIGIFT	14-27	0.83
	**SSAIVSVSNLDDNKGLG**	**43-59**	**LDDNKGLGKAHA**	**52-63**	**0.764**	**TDNSKKDITDQVTW**	**27-40**	**0.77**
						**NKGLGKAHAVGDTT**	**55-68**	**0.76**
LigB6	NPVNPSLAKGLT	6-17	**AQKNQGNAYGAA**	**53-64**	**0.973**	**ISNAQKNQGNAYGA**	**50-63**	**0.87**
	**YSDNSNKDIT**	**26-35**	TFGKVSSPVSTL	83-94	0.847	**GIYSDNSNKDITSA**	**24-37**	**0.82**
	SSDS	42-45	**FSATGIYSDNSN**	**20-31**	**0.503**	QKFSATGIYSDNSN	18-31	0.78
	**NAQKNQG**	**52-58**	VTWFSSDSSIAT	38-49	0.469	KDITSAVTWFSSDS	32-45	0.77
			TSIQINPVNPSL	1-12	0.357	QGNAYGAATGATDI	57-70	0.75
LigB7	TPAAASKAKGLT	6-17	ITNTSGSKGITN	50-61	0.999	ITPAAASKAKGLTE	5-18	0.89
	**FTDNSNSDIT**	**26-35**	**FTDNSNSDITNQ**	**26-37**	**0.992**	**TDNSNSDITNQVTW**	**27-40**	**0.83**
	NTSGSKGI	52-59				ASKAKGLTERFKAT	10-23	0.82
						SNTDIAEITNTSGS	43-56	0.79
						NQVTWNSSNTDIAE	36-49	0.76
LigB8	**TPINPSVAKGLI**	**6-17**	VNNVTGSVTTVA	50-61	0.989	VQDVTALATWSSSN	31-44	0.89
	**YTDHSVQDVT**	**26-35**	**IAVTPINPSVAK**	**3-14**	**0.979**	**FKATGTYTDHSVQD**	**20-33**	**0.84**
	SSSNPRKAMV	41-50	**ATGTYTDHSVQD**	**22-33**	**0.911**	KGLIRQFKATGTYT	14-27	0.83
						GNTNIKATIDSISG	63-76	0.82
						SVTTVATGNTNIKA	56-69	0.80
						NPRKAMVNNVTGSV	44-57	0.78
						**PINPSVAKGLIRQF**	**7-20**	**0.77**
LigB9	**PTINSITHGLT**	**7-17**	**FSDKSTQNLTQL**	**26-37**	**0.986**	TQNLTQLVTWISSD	31-44	0.88
	**FSDKSTQNLT**	**26-35**	IENTSGKKGIAT	50-61	0.899	SDPSKIEIENTSGK	43-56	0.85

	ISSDPSKIEIENTSGKK GIA	41-60	IQSSPIPITVTD	77-89	0.595	**PTINSITHGLTKQF**	**7-20**	**0.82**
	L	65	**EITPTINSITHG**	**4-15**	**0.497**	**KATGIFSDKSTQNL**	**21-34**	**0.79**
	FI	76-77				THGLTKQFKATGIF	13-26	0.77
						NTSGKKGIATASKL	52-65	0.76
LigB10	PSSSSIAKGLTQ	7-18	VAPINNAANEKG	47-58	1	QQFKAIGTFIDGSE	18-31	0.90
	**FIDGSEQEI**	**26-34**	**GSEQEITNLVTW**	**29-40**	**0.741**	**ITNLVTWYSSKSDV**	**34-47**	**0.75**
	YSSKS	41-45						
	AANEKGLA	53-60						
	INPVNNNIAKGLT	5-17	**ISNSTETKGKAT**	**50-61**	**0.97**	QQYTALGVYSDSTI	18-31	0.90
LigB11	**YSDSTIQDIS**	**26-35**	**YSDSTIQDISDS**	**26-37**	**0.865**	ALQIGNSKITATYN	62-75	0.85
	SNS	43-45	IKINPVNNNIAK	3-14	0.853	SSNSSSISISNSTE	42-55	0.84
	**STETKGK**	**53-59**				**SDSTIQDISDSVTW**	**27-40**	**0.82**
						**NSTETKGKATALQI**	**52-65**	**0.79**
						AKGLTQQYTALGVY	13-26	0.77
LigB12	**ISPINTNINTTVS**	**5-17**	**SPINTNINTTVS**	**6-17**	**0.936**	KADLTSSVTWSSSN	31-44	0.91
	**SAGTKADL**	**27-34**	VTGIASGNPTII	60-71	0.916	VTGIASGNPTIIAT	60-73	0.90
	SNQ	43-45	QAKVSNASETKG	47-58	0.339	**FFAVGTYSAGTKAD**	**20-34**	**0.85**
	ETK	55-57	**GTYSAGTKADLT**	**24-35**	**0.327**	KVSNASETKGLVTG	49-62	0.84
						**PINTNINTTVSKQF**	**7-20**	**0.76**

Supplementary Material

Table S4: List of predicted CTL epitopes

Domain	IEDB	NetMHC 4.0	ProPred
LigA7	FTDNSNSDI	FTDNSNSDI	FTDNSNSDI
	GTVKVTASM	GTVKVTASM	GTVKVTASM
	LTVSNTNAK	SMGGIEDSV	SMGGIEDSV
	STLKQGTVK	VEIQITPAA	VEIQITPAA
	LTVSNTNAK	LTVSNTNAK	LTVSNTNAK
			STLKQGTVK
LigA8		ATLEKLSGK	ATLEKLSGK
		TWKSSSKAL	TWKSSSKAL
		EVSPTRASI	EVSPTRASI
		KSSSKALSM	KSSSKALSM
		KNITEQVTW	KNITEQVTW
	QVTWKSSSK		QVTWKSSSK
	SIAKGMTQK		SIAKGMTQK
	GIFTDHSKK		GIFTDHSKK
LigA9	ATISNTKGY	ATISNTKGY	ATISNTKGY
	SVATISNTK	SVATISNTK	SVATISNTK
		TLGNVSSQV	TLGNVSSQV
		KDITSAVTW	KDITSAVTW
		WHSSNNSVA	WHSSNNSVA
		SSKDITSAV	SSKDITSAV
		GTVDIKATL	GTVDIKATL
		YSDNSSKDI	YSDNSSKDI
LigA10	SSANATFQV	SSANATFQV	SSANATFQV
	GVFTDNSTK	GVFTDNSTK	GVFTDNSTK
	KISNATGSK	KISNATGSK	KISNATGSK

	ATFQVTPAK	ATFQVTPAK	ATFQVTPAK
		ALSKGTSHI	ALSKGTSHI
		GTSHISATL	GTSHISATL
		SISSANATF	SISSANATF
		TWKSSNTAY	TWKSSNTAY
	KSSNTAYAK		KSSNTAYAK
LigA11	FAKGNSYQF	FAKGNSYQF	FAKGNSYQF
	SVLNVTPAL	SVLNVTPAL	SVLNVTPAL
	KIASVENTF	KIASVENTF	KIASVENTF
	ISFAKGNSY	ISFAKGNSY	ISFAKGNSY
	VTWSSSNPK	VTWSSSNPK	VTWSSSNPK
	YQFKATGIY	YQFKATGIY	YQFKATGIY
	EVIPNNISF	EVIPNNISF	EVIPNNISF
		YTDHSEADI	YTDHSEADI
		ITAKLSDTV	ITAKLSDTV
		DTVSGSSVL	DTVSGSSVL
		NTFGSAGLV	NTFGSAGLV
LigA12	YSDQSTKDL	YSDQSTKDL	YSDQSTKDL
	TPGKKGLAF	TPGKKGLAF	TPGKKGLAF
	FSSNPSSVV	FSSNPSSVV	FSSNPSSVV
	HTQSSYTPV	HTQSSYTPV	HTQSSYTPV
	KQFSAIGTY	KQFSAIGTY	KQFSAIGTY
	YDHHTQSSY	YDHHTQSSY	YDHHTQSSY
	RYIMITPSY	RYIMITPSY	RYIMITPSY
		YIMITPSYA	YIMITPSYA
		ELGEPDITV	ELGEPDITV
		MITPSYAGI	MITPSYAGI
	VVIENTPGK		VVIENTPGK
	GTYSDQSTK		GTYSDQSTK
LigA13	KTKGSTHQF	KTKGSTHQF	KTKGSTHQF
	AEIDLTELV	AEIDLTELV	AEIDLTELV

		HQFKATGKF	HQFKATGKF
		WSSSNPTVV	WSSSNPTVV
	FENGAEIDL		FENGAEIDL
	SSNPTVVSI		SSNPTVVSI
	ISSSIDFEV		ISSSIDFEV
	TISLSSISK		TISLSSISK
LigB1	IANGTSTTL	IANGTSTTL	IANGTSTTL
	TTLEVTAIF	TTLEVTAIF	TTLEVTAIF
		IKAEYNGLY	IKAEYNGLY
LigB2	HIRASFQSK	HIRASFQSK	HIRASFQSK
	RQFSAIGIF	RQFSAIGIF	RQFSAIGIF
	EEMTVGDAV	EEMTVGDAV	EEMTVGDAV
		GTAHIRASF	GTAHIRASF
		LPKGTNRQF	LPKGTNRQF
		HQDISNDPL	HQDISNDPL
		ISNDPLIVW	ISNDPLIVW
	FSDGSHQDI		FSDGSHQDI
LigB3		KQKLIATGI	KQKLIATGI
		IPLGKKQKL	IPLGKKQKL
		YSDNSNRDI	YSDNSNRDI
		TADTGIVTI	TADTGIVTI
LigB4		FTDNSNSDI	FTDNSNSDI
		IQGSTDFTV	IQGSTDFTV
LigB5	TSIEVSPVL		TSIEVSPVL
	TTITATLGK		TTITATLGK
LigB6	AQKNQGNAY	AQKNQGNAY	AQKNQGNAY
	STLSVTAAK	STLSVTAAK	STLSVTAAK
		QKFSATGIY	QKFSATGIY
		KVSSPVSTL	KVSSPVSTL
		KDITSAVTW	KDITSAVTW
		GATDIKATF	GATDIKATF

LigB7	FTDNSNSDI	FTDNSNSDI	FTDNSNSDI
		EISAALGSI	EISAALGSI
		ERFKATGIF	ERFKATGIF
		VEIQITPAA	VEIQITPAA
LigB8	RQFKATGTY	RQFKATGTY	RQFKATGTY
	MVNNVTGSV	MVNNVTGSV	MVNNVTGSV
	SVLNVTPAL	SVLNVTPAL	SVLNVTPAL
	ATWSSSNPR	ATWSSSNPR	ATWSSSNPR
	YTDHSVQDV	YTDHSVQDV	YTDHSVQDV
	HSVQDVTAL	HSVQDVTAL	HSVQDVTAL
LigB9	FSDKSTQNL	FSDKSTQNL	FSDKSTQNL
	TSIEITPTI	TSIEITPTI	TSIEITPTI
	QSSPIPITV	QSSPIPITV	QSSPIPITV
	FIQSSPIPI	FIQSSPIPI	FIQSSPIPI
	YKFIQSSPI	YKFIQSSPI	YKFIQSSPI
		TINSITHGL	TINSITHGL
		KQFKATGIF	KQFKATGIF
		ITHGLTKQF	ITHGLTKQF
		SNIKAVYKF	SNIKAVYKF
		EITPTINSI	EITPTINSI
		KSTQNLTQL	KSTQNLTQL
	SSNIKAVYK		SSNIKAVYK
LigB10	GSSDIYAIY	GSSDIYAIY	GSSDIYAIY
	FIDGSEQEI	FIDGSEQEI	FIDGSEQEI
	LSIGSSDIY	LSIGSSDIY	LSIGSSDIY
	NEKGLATAL	NEKGLATAL	NEKGLATAL
	YAIYNSISS	YAIYNSISS	YAIYNSISS
	QEITNLVTW	QEITNLVTW	QEITNLVTW
		EITNLVTWY	EITNLVTWY
		DIYAIYNSI	DIYAIYNSI
		SSNKINFNV	SSNKINFNV

		QQFKAIGTF	QQFKAIGTF
LigB11	YSDSTIQDI	YSDSTIQDI	YSDSTIQDI
	QQYTALGVY	QQYTALGVY	QQYTALGVY
	WSSSNSSSI	WSSSNSSSI	WSSSNSSSI
		TIQDISDSV	TIQDISDSV
		ETKGKATAL	ETKGKATAL
	KITATYNSI		KITATYNSI
LigB12	KQFFAVGTY	KQFFAVGTY	KQFFAVGTY
		TIIATYGSV	TIIATYGSV
		TVSKQFFAV	TVSKQFFAV
		ETKGLVTGI	ETKGLVTGI
		YSAGTKADL	YSAGTKADL

Supplementary Material

Table S5: List of predicted HTL epitopes

Domain	ProPred-I	NetMHC 2.3	IEDB
LigA7	VEIQITPAA	VEIQITPAA	
	ILTVSNTNA	ILTVSNTNA	
	VKVTASMGG	VKVTASMGG	
	LVEIQITPAA	LVEIQITPAA	
	ILTVSNTNA	ILTVSNTNA	
	IQITPAAAS	IQITPAAAS	
LigA8	VTWKSSSK	VTWKSSSK	
	ISITATLEK	ISITATLEK	
	IEVSPTRAS	IEVSPTRAS	
LigA9	ITSAVTWHS	ITSAVTWHS	
	ITSAVTWHSSNNS	ITSAVTWHSSNNS	
	VDIKATLGNVS	VDIKATLGNVS	
	IKATLGNVSSQVS	IKATLGNVSSQVS	
	SNNSVATISNTKG	SNNSVATISNTKG	
LigA10	IVLNPTSSH	IVLNPTSSH	IVLNPTSSH
	WKSSNTAYA	WKSSNTAYA	
	IEIVLNPTS	IEIVLNPTS	
	IVLNPTSSHKA	IVLNPTSSHKA	
	LGSISSANA	LGSISSANA	
LigA11	SVLNVTPALL	SVLNVTPALL	

	VIPNNISFA	VIPNNISFA	
	IEVIPNNIS	IEVIPNNIS	
	YQFKATGIY	YQFKATGIY	
	LVSTTNIGS	LVSTTNIGS	
LigA12	YIMITPSYA	YIMITPSYA	YIMITPSYA
	IMITPSYAG	IMITPSYAG	IMITPSYAG
	WFSSNPSSV	WFSSNPSSV	
	QSSYTPVTVTES	QSSYTPVTVTES	
	TVVFYDHHTQS	TVVFYDHHTQS	
	VTWFSSNPSSV	VTWFSSNPSSV	
LigA13	TVVSISNVDD	TVVSISNVDD	
	LVTWSSSNPT	LVTWSSSNPT	
	IVNITISLS	IVNITISLS	
	VNITISLSS	VNITISLSS	
	ITISLSSIS	ITISLSSIS	
LigB1	VVTIQGNRV	VVTIQGNRV	
	VTIQGNRVR	VTIQGNRVR	
	IQGNRVRGI	IQGNRVRGI	
LigB3	VKLIVTPAA	VKLIVTPAA	VKLIVTPAA
	IVTISASSE	IVTISASSE	
	IWNSSNSTIA	IWNSSNSTIA	
	IQVTSNNPNI	IQVTSNNPNI	
	VKLIVTPAAL	VKLIVTPAAL	
	GIVTISASSE	GIVTISASSE	

	IVTISASSEN	IVTISASSEN	
LigB5	WLTVVPAVL	WLTVVPAVL	
	WNSSSAIVS	WNSSSAIVS	
	VLPSIAKGL	VLPSIAKGL	
LigB6	IQINPVNPS	IQINPVNPS	IQINPVNPS
	ITSAVTWFS	ITSAVTWFS	
	WFSSDSSIA	WFSSDSSIA	
	VNPSLAKGL		VNPSLAKGL
LigB7	ILKVTPAQL	ILKVTPAQL	ILKVTPAQL
	VEIQITPAA	VEIQITPAA	
	VILKVTPAQ	VILKVTPAQ	
	IKSSKVILK	IKSSKVILK	
	IQITPAAAS	IQITPAAAS	
LigB8	VLNVTPALL	VLNVTPALL	
	LIRQFKATGTY	LIRQFKATGTY	
	MVNNVTGSV	MVNNVTGSV	
	VTPINPSVATWSS	VTPINPSVATWSS	
LigB9	AVYKFIQSSPIPITV	AVYKFIQSSPIPITV	AVYKFIQSSPIPITV
	FIQSSPIPI	FIQSSPIPI	
	SIEITPTINS	SIEITPTINS	
	WISSDPSKI	WISSDPSKI	
LigB10	SDIYAIYNSISSNKI	SDIYAIYNSISSNKI	SDIYAIYNSISSNKI
	SSDIYAIYNSISSNK	SSDIYAIYNSISSNK	SSDIYAIYNSISSNK
	LVTWYSSKS	LVTWYSSKS	

	IYAIYNSIS	IYAIYNSIS	
	YAIYNSISS	YAIYNSISS	
	FKAIGTFID	FKAIGTFID	
	ITISPSSSS	ITISPSSSS	
LigB11	VTWSSSNSS	VTWSSSNSS	
	IKINPVNNN	IKINPVNNN	
LigB12	FFAVGTYSA	FFAVGTYSA	
	VTWSSSNQS	VTWSSSNQS	
	ISPINTNINTTV	ISPINTNINTTV	

Figure legends

Figure S1: Separate RMSD plot for the vaccine construct and TLR4 at 100ns.

Figure S2: RMSF plot for vaccine construct only at 50ns of MDS.

Figure S3: Separate Rg plot for TLR4 and vaccine construct at 100ns of MDS.

Figure S1

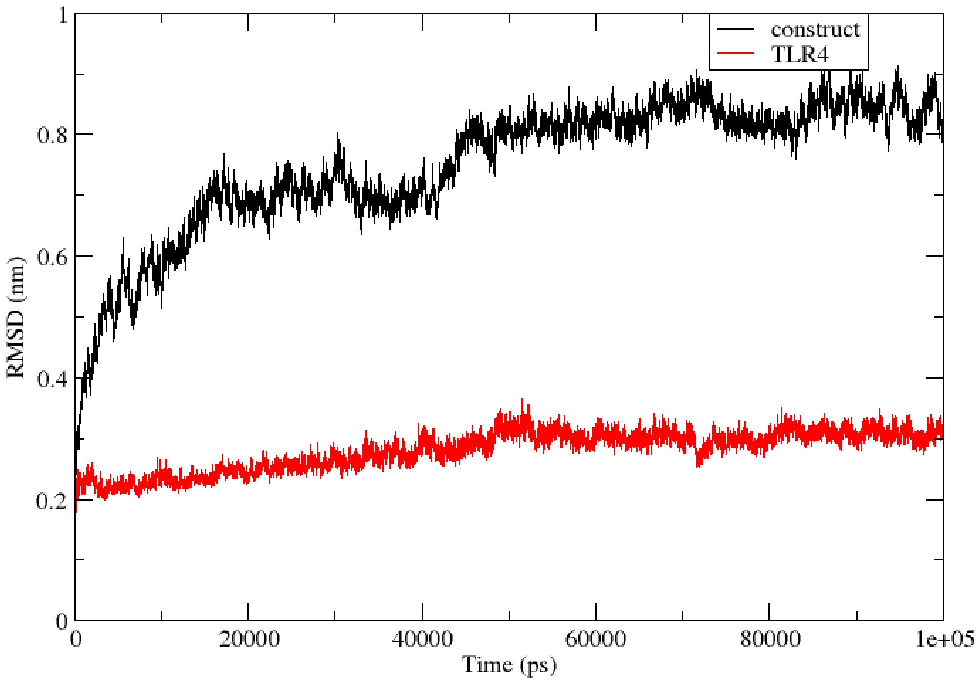
construct
TLR4
RMSD (nm)
0
0.2
0.4
0.6
0.8
1
0
20000
40000
60000
80000
1e+05
Time (ps)

Figure S2

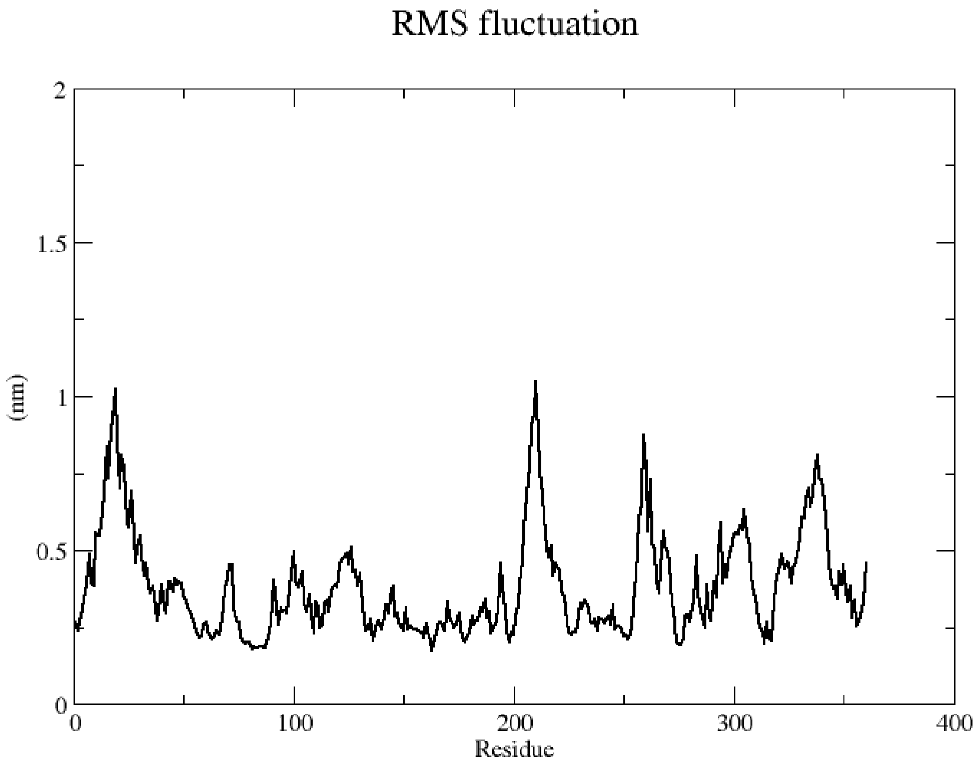

Figure S3

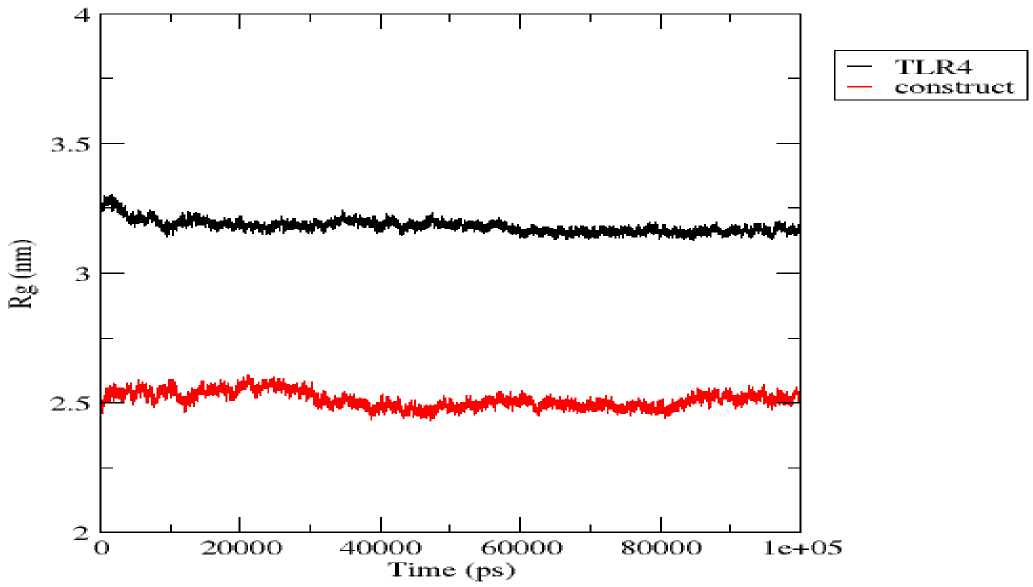
4
3.5
3
2.5
2
Rg (nm)
0
20000
40000
60000
80000
1e+05
Time (ps)
TLR4
construct
